# The importin beta superfamily member RanBP17 exhibits a role in cell proliferation and is associated with improved survival of patients with HPV+ HNSCC

**DOI:** 10.1186/s12885-022-09854-0

**Published:** 2022-07-18

**Authors:** Robert Mandic, André Marquardt, Philip Terhorst, Uzma Ali, Annette Nowak-Rossmann, Chengzhong Cai, Fiona R. Rodepeter, Thorsten Stiewe, Bernadette Wezorke, Michael Wanzel, Andreas Neff, Boris A. Stuck, Michael Bette

**Affiliations:** 1 Department of Otorhinolaryngology, Head and Neck Surgery, University Hospital Giessen and Marburg, Campus Marburg, Philipps-Universität Marburg, 3. BA, +3/08070, Marburg, Germany; 2 Comprehensive Cancer Center Mainfranken, University Hospital Würzburg, Würzburg, Germany; 3 Institute of Pathology, University of Würzburg, Würzburg, Germany; 4 Bavarian Cancer Research Center (BZKF), Würzburg, Germany; 5 Institute for Pharmaceutical Technology & Biopharmacy, Philipps-Universität Marburg, Marburg, Germany; 6 Department of Molecular Neuroscience, Institute of Anatomy and Cell Biology, Philipps-Universität Marburg, Marburg, Germany; 7 Institute of Pathology, University Hospital Giessen and Marburg, Campus Marburg, Marburg, Germany; 8 Institute of Molecular Oncology, Universities of Giessen and Marburg Lung Center (UGMLC), Member of the German Center for Lung Research (DZL), Philipps-Universität Marburg, Marburg, Germany; 9 Department of Oro- and Maxillofacial Surgery, University Hospital Giessen and Marburg, Campus Marburg, Philipps-Universität Marburg, Marburg, Germany

**Keywords:** RanBP17, circRanBP17, HNSCC, HPV, Proliferation, Survival

## Abstract

**Background:**

More than twenty years after its discovery, the role of the importin beta superfamily member Ran GTP-binding protein (RanBP) 17 is still ill defined. Previously, we observed notable *RanBP17* RNA expression levels in head and neck squamous cell carcinoma (HNSCC) cell lines with disruptive *TP53* mutations.

**Methods:**

We deployed HNSCC cell lines as well as cell lines from other tumor entities such as HCT116, MDA-MB-231 and H460, which were derived from colon, breast and lung cancers respectively. RNAi was used to evaluate the effect of *RanBP17* on cell proliferation. FACS analysis was used for cell sorting according to their respective cell cycle phase and for BrdU assays. Immunocytochemistry was deployed for colocalization studies of RanBP17 with Nucleolin and SC35 (nuclear speckles) domains. TCGA analysis was performed for prognostic assessment and correlation analysis of *RanBP17* in HNSCC patients.

**Results:**

RNAi knockdown of *RanBP17*, significantly reduced cell proliferation in HNSCC cell lines. This effect was also seen in the HNSCC unrelated cell lines HCT116 and MDA-MB-231. Similarly, inhibiting cell proliferation with cisplatin reduced RanBP17 in keratinocytes but lead to induction in tumor cell lines. A similar observation was made in tumor cell lines after treatment with the EGFR kinase inhibitor AG1478. In addition to previous reports, showing colocalization of RanBP17 with SC35 domains, we observed colocalization of RanBP17 to nuclear bodies that are distinct from nucleoli and SC35 domains. Interestingly, for HPV positive but not HPV negative HNSCC, TCGA data base analysis revealed a strong positive correlation of *RanBP17* RNA with patient survival and *CDKN2A*.

**Conclusions:**

Our data point to a role of RanBP17 in proliferation of HNSCC and other epithelial cells. Furthermore, RanBP17 could potentially serve as a novel prognostic marker for HNSCC patients. However, we noted a major discrepancy between RanBP17 RNA and protein expression levels with the used antibodies. These observations could be explained by the presence of additional *RanBP17* splice isoforms and more so of non-coding *circular RanBP17* RNA species. These aspects need to be addressed in more detail by future studies.

**Supplementary Information:**

The online version contains supplementary material available at 10.1186/s12885-022-09854-0.

## Background

Head and neck squamous cell carcinomas (HNSCC) are the most frequent cancers of the upper aerodigestive tract [1, 2]. Virtually all HNSCC present with inactivation of the tumor suppressor protein p53, either as a result of mutations in the *TP53* gene or due to inactivation of the p53 protein as a consequence of high-risk human papillomavirus (HPV) infection. Reduction or loss of p53 function is a major driving force for tumor development, progression and therapy resistance in HNSCC and other cancers. Early it was recognized that nuclear localization of p53 is required for its proper function emphasizing its role as a transcription factor [3]. Studies by several groups, including our own, observed that disrupting *TP53* mutations such as those leading to a premature stop codon that result in truncated and cytoplasmically sequestered p53 proteins are distinct from non-disrupting *TP53* mutations, like the typical hot spot mutations found in the DNA binding domain of the protein [4, 5]. Specifically, HNSCC cells carrying such truncated, cytoplasmically sequestered mutant p53 proteins were significantly more resistant to the chemotherapeutic agent cisplatin (CDDP). Consistent with these observations, HNSCC patients with this type of *TP53* mutation exhibit a significantly worse prognosis [5]. Moreover, HNSCC cells with truncated, cytoplasmic p53 appear to exhibit stem-cell like features such as ABC (ATP-binding Cassette) transporter upregulation, higher metabolism and glutathione levels [6]. Analyzing, the same micro array data as in our previous study [6], we observed upregulation of *RanBP (Ran GTP-binding protein) 17* transcript levels in cell lines with cytoplasmic mutant p53. RanBP17 is an ill-defined member of the importin beta superfamily (karyopherin). The gene encoding *RanBP17* initially was identified due to sequence homology to its homologue *RanBP16* that was isolated by affinity chromatography after binding to immobilized RanGTP. Both homologues, *RanBP16* and *RanBP17*, exhibited the highest homology to exportins such as exportin 4 and exportin 1 (CRM1, XPO1) [7]. Notably, *RanBP17* was independently discovered while investigating genes at the breakpoint region t(5;14) (q34;q11) that is found in a significant number of acute lymphoblastic leukemias [8]. Furthermore, it was noted that *RanBP17* presumably appeared late in evolution and likely is restricted to vertebrates, whereas its homologue *RanBP16* is found widely distributed in many higher eukaryotes [7]. However, until now, it could not be unequivocally determined if RanBP17 acts as an exportin or an importin. Although binding of RanBP17 to the basic helix loop helix transcription factor E12 was demonstrated in a yeast two hybrid binding assay [9], the exact cargoes of RanBP17 still need to be identified and validated. Proteins involved in the nucleocytoplasmic transport of the cell [10] have been implicated in tumor progression [11], were found to be secreted by tumors thereby acting as potential tumor markers [12] and are considered as potential targets for tumor therapy [13–15]. The present study aimed to evaluate the potential role of RanBP17 in HNSCC disease.

## Methods

### Tissues and cell lines

Tumor tissues (Supplementary Table S1) were used for Western blot analysis according to the requirements and guidelines of the local ethics committee (ethic code: 149/07; Ethics Committee, Department of Medicine, Philipps-Universität Marburg, Germany). All methods were carried out in accordance with relevant guidelines and regulations. The tissue samples used for the study were old (1998–2004) archived anonymized tissues. HNSCC cell lines were kindly provided by Dr. T. Carey (University of Michigan, Ann Arbor, MI) and Dr. R. Grènman (University of Turku, Turku, Finland) [4, 16]. Cell line authentication was performed for the key HNSCC cell lines UM-SCC-3 and UT-SCC-26A as well as UM-SCC-4, UM-SCC-22B, UM-SCC-27 and UT-SCC-24A cells according to the published genotype [16] or by validating the known *TP53* mutations as reported for these cell lines [4]. Furthermore, the colon cancer derived cell line HCT116, the breast cancer derived cell line MDA-MB-231 and the lung cancer derived cell line H460 were included as well for representation of other major solid cancer types. Other cells and cell lines as listed in Supplementary Table S2 were solely used for the purpose of Western blot analysis screening for RanBP17 expression and except for Normal Human Epidermal Keratinocytes (NHEK) were not used in further experiments. CRISPR/Cas9 *TP53* knock out HCT116 and H460 cell lines were generated and validated as previously described by Wanzel et al. [17]. MDA-MB-231 *TP53* knockout clones were generated in the same manner as described for the HCT116 and H460 cells and successful *TP53* knockout was validated by sequence and Western blot analysis. *TP53* knockout cell lines were included since *RanBP17* was initially found differentially regulated in cancer cells with disruptive *TP53* mutations. All cells, except NHEK and Human Umbilical Vein Endothelial Cells (HUVEC), were cultured under standard conditions (37 °C, 5% CO_2_) in Dulbecco’s Modified Eagle Medium (DMEM) containing 10% fetal bovine serum (FBS), 100 U/ml penicillin, 100 µg/ml streptomycin, 50 µg/ml gentamicin, 2 mmol/L L-glutamine. NHEK (cat#: C-12007, PromoCell GmbH, Heidelberg, Germany) were grown in Keratinocyte Growth Medium 2 (cat#: C-20011, PromoCell GmbH). HUVEC cells were cultured in EGM^TM^-2 Endothelial Cell Growth Medium-2 BulletKit™ (Lonza).

### Flow cytometry

The two HNSCC cell lines, UM-SCC-3 and UT-SCC-26A, were sorted according to their cell cycle phase using a modified protocol from Arndt-Jovin and Jovin [18]. Cells were grown in eight 10 cm cell culture dishes until reaching 80% confluence. Cells were then trypsinized and collected in a 50 ml Falcon tube by passing them through a cell filter (Falcon® 100 μm Cell Strainer, cat# 352360). After pelleting cells at 300 x g for 10 min they were resuspended in 10 ml HBSS medium containing 2% FBS and counted. After adjusting the cell number to 1 × 10^6^ /ml, Hoechst 33342 (stock: 1 mg/ml) was added to a final concentration of 10 µg/ml followed by incubation in a water bath for 90 min at 37 °C. Cells were pelleted and 90% of the medium supernatant was removed followed by resuspension of the cells in the remaining volume resulting in a cell concentration of 10 × 10^6^ /ml. After incubation at 37 °C, cells were kept at 4 °C during all following steps. FACS tubes used for collection of sorted cells were previously filled with FCS and incubated for 1 h at 37 °C to coat the inner side of the tube aiming to prevent sticking of cells to the tube surface during cell sorting. After incubation, the FCS was removed leaving 500 µl FCS in the tube. Sorting of cells was performed with the MoFlow Astrios System (Beckman Coulter, Software: Summit V6.2.7.16492) at the Flow Cytometry Core Facility (Faculty of Medicine, Philipps-Universität Marburg, Director: Dr. C. Brendel). Cells were gated according to the G1/G0, S and G2/M phases of the cell cycle. In addition, cells from all 3 gates (all phases) were sorted into a single tube for reference, representing the whole population. Approximately 1 × 10^6^ cells or more were collected for each cell cycle phase and exact cell numbers were documented. After sorting, cells were pelleted for 10 min at 300 x g (4 °C) and subsequently used for RNA and protein extraction. Same absolute amounts of “protein or RNA” or “protein or RNA levels adjusted to the respective cell number” were evaluated in the Western blot and RT-qPCR analyses. For the bromodeoxyuridine (BrdU) assay, the HNSCC cell lines UM-SCC-3 and UT-SCC-26A were treated with 20 µg/ml of the EGFR kinase inhibitor AG1478 (Calbiochem®). The BrdU assay was performed according to a modified protocol from BioLegend® (Rev. 05102016, San Diego, CA) BrdU (BD Pharmingen™, Cat No. 550891) was added to a final concentration of 10 µmol/L to the culture medium and incubation of cells (37 °C, 5% CO_2_) was continued for 2 more hours. Cells were harvested for FACS (70% ice cold ethanol) and Western blot analyses. Western blot analysis was performed as described below. Cells to be used for FACS were centrifuged for 10 min at 300 x g and washed in 0.5% BSA / PBS. The resulting pellet was incubated for 20 min at room temperature in 1 ml of 2 mol/L HCl, washed again and incubated for 2 min in 0.1 mol/L sodium tetraborate (Na_2_B_4_O_7_, pH 8.5). After repeating the washing step, the pellet was resuspended in 50 µl dilution solution (0.5% BSA and 0.5% Tween-20 in PBS). Fluorescently labeled mouse anti BrdU antibody (Alexa Fluor® 647 mouse anti-BrdU, 5 µl, Cat#: 560209, BD Pharmingen; Alexa Fluor® 488 BrdU Monoclonal Antibody (MoBU-1), 5 µl, Cat#: B35130, ThermoFisher Scientific/Invitrogen; BD Horizon™ V450 mouse anti-BrdU, 2.5 µl, Cat#: 560810) was added and cells were incubated for 20 min. Finally, cells were washed and resuspended in 500 µl propidium iodide solution (10 µg/ml in PBS) followed by flow cytometry. FACS data was analyzed with the FlowJo™ software (version 7.6.5, Tree Star Inc., Ashland, OR).

### Western blot analysis

SDS PAGE and Western blot analyses were performed under standard conditions [4]. Rabbit polyclonal antibodies specific for RanBP17 were purchased from Biorbyt Ltd. (orb226830, directed against amino acids 50–70 of human RanBP17, NP_075048.1, Cambridge, UK) and GeneTex, Inc. (GTX70420, directed against amino acids 946–1088 of human RanBP17, NP_075048.1, Irvine, CA). GAPDH (clone 0411, sc-47724), PCNA (clone PC10, sc-56), CDK1 (Cdc2 p34, clone 17, sc-54) and Cyclin-B1 (clone GNS1, sc-245) specific mouse monoclonal antibodies and the beta-Tubulin (sc-9104) specific rabbit polyclonal antibody were from Santa Cruz Biotechnology, Inc. (Dallas, TX). All secondary HRP-coupled antibodies directed against mouse (sc-2096) or rabbit (sc-2004) IgG were from Santa Cruz Biotechnology, Inc. The mouse monoclonal antibody directed against β-Actin was purchased from Sigma-Aldrich, Inc. (cat#: A5316; clone AC-74; Saint Louis, MO). Uncropped Western blot images are shown in Supplementary Fig. S1.

### RNAi knockdown

Small interfering RNAs specific for *RanBP17* (siGENOME SMARTpool®, Cat#: M-015496-00, Human RanBP17, NM_022897 and ON-TARGETplus SMARTpool, Cat#: L-015496-02) were obtained from Dharmacon (Thermo Fisher Scientific - Dharmacon Products, Lafayette, CO). Non-targeting small RNAs (ON-TARGETplus Non-targeting Pool, D-001810-10-20) were used as a negative control as described previously [6]. Cells were transfected with the respective small interfering or non-targeting RNA using HiPerFect (Qiagen, Hilden, Germany) as a transfection reagent according to the manufacturer’s protocol. Transfected cells were incubated for 72 h at standard culture conditions and subsequently used in downstream applications such as RT-qPCR and XTT proliferation assays.

### XTT (2,3-Bis-(2-methoxy-4-nitro-5-sulfophenyl)-2*H*-tetrazolium-5-carboxanilide) viability assay

UM-SCC-3, -4, -27, UT-SCC-26A, HCT116^p53 wt/wt^, HCT116^p53 −/−^, H460^p53 wt/wt^, H460^p53 −/−^, MDA-MB-231^p53 mut/mut^ and MDA-MB-231^p53 −/−^ cells were grown until reaching 80% confluence. After washing in PBS (w/o Ca^++^ & Mg^++^), cells were detached by trypsin and counted. The cell number was adjusted to 100 cells/µl. Fifty µl of the cell suspension (5 × 10^3^ cells) was added per well into a 96 well cell culture plate followed by incubation for 24 h. Transfection of cells with *RanBP17* siRNA or non-targeting RNA was performed as described above and incubation was continued for 72 h. Experiments were performed at least in triplicate. In a preliminary experiment using UM-SCC-3 cells treated with *RanBP17* siRNA or non-targeting RNA, CDDP (#20407-2; Sigma-Aldrich, Inc.) was added to a final concentration of 6.25, 12.5, 25, 50 or 100 µmol/L and incubation was continued for 24 more hours. The XTT viability assay (Cell Proliferation Kit II (XTT), Cat. No. 11 465 015 001, Roche Diagnostics GmbH, Mannheim, Germany) was performed according to the manufacturer’s instructions. Absorbance was measured with a DTX880 microplate reader (Beckman Coulter, Inc., Fullerton, CA) at 450 and 620 (reference wavelength) nm.

### Quantification of RNA

Whole cellular RNA was isolated with the Trizol method (Molecular Research Center, Inc., Cincinnati, OH) and the RNeasy Mini kit (Qiagen), according to the manufacturer’s protocol. RNA quantification and quality control was done with the Nanodrop and Experion systems. Gene expression patterns of HNSCC cell lines were determined by micro array analysis (GeneChip® Human Gene 1.0 ST Array-System, Affymetrix Inc., Santa Clara, CA) as previously reported [6]. Validation of *RanBP17* gene expression levels was performed by RT-qPCR. Total RNA was reversely transcribed into cDNA using the Transcriptor First Strand cDNA Synthesis Kit (Roche). Absolute quantitative RT-PCR was performed with the *RanBP17* (REFSeq: NM_022897.4, GenBank) specific primers 5’-CCCAAGCAGGAGGTC-3’ (forward, nt 3240–3254) and 5’-ATGGTCAGAAAAGTCGG-3’ (reverse complement, nt 3421–3437) to determine the copy numbers of *RanBP17* RNA. For this, a standard curve of *RanBP17* templates with known copy numbers were generated (efficiency = 0.98; amplification rate = 1.962). The amount of amplified RNA in each probe was normalized against the ribosomal protein S18 (RPS18; PrimePCR™ PreAmp for Probe Assay: RPS18, Human, Bio-Rad Laboratories GmbH, Feldkirchen, Germany). Quantitative RT-qPCR was performed with the same primers as used for absolute RT-qPCR. Samples were amplified with the Power SYBR Green PCR Master Mix (Applied Biosystems) and run in triplicate (ABI PRISM 7900HT System, Applied Biosystems and QuantStudio 5, Thermo Fisher Scientific).

### Incubation of keratinocytes and tumor cell lines with CDDP

In one preliminary experiment, keratinocytes were incubated for 24 h at 37 °C, 5% CO_2_ in the presence of 0, 5 and 50 µmol/L CDDP. Cells were subsequently trypsinized and collected by centrifugation at 300 x g for 10 min followed by protein extraction and Western blot analysis as described above. The experiment was repeated four times for the purpose of RNA extraction and RT-qPCR analysis. Similarly, two tumor cell lines, HCT116^p53 wt/wt^ and UM-SCC-3, were exposed for 24 h to different CDDP levels (0.78, 1.56, 3.13, 6.25, 12.5, 25, 50 and 100 µmol/L) with subsequent RNA extraction and RT-qPCR using 11 different RanBP17 specific primer pairs (see Results below).

### Immunocytochemistry

Cells were grown on coverslips in six well tissue culture dishes and cultured as described above. After reaching 50% confluence, cells were rinsed with PBS and fixed in cold (-20 °C) methanol for 5 min. Immunocytochemistry was performed as previously described [19]. Primary antibodies were directed against RanBP17 (HPA029568, Atlas Antibodies, Bromma, Sweden), SC35 (clone SC-35, Sigma-Aldrich, Inc.) or Nucleolin (clone ZN004, Thermo Fisher Scientific, Rockford, IL). The blocking peptide APrEST73986 (Atlas Antibodies) was deployed to validate specificity of the RanBP17 antibody HPA029568. Secondary antibodies for immunocytochemistry analysis were Alexa Fluor 488 and Alexa Fluor 647-coupled anti rabbit or anti mouse IgG directed antibodies (Thermo Fisher Scientific). Microscopic analysis was done with a Zeiss Axio Imager.M2 (Carl Zeiss Microscopy Deutschland GmbH, Oberkochen, Germany).

### Statistical analysis

Statistic differences in: (i) the level of cell viability after *RanBP17* RNAi treatment compared to the respective control (NT, non-target RNA) and (ii) between the AG1478 and DMSO groups at different cell cycle phases were calculated by an unpaired two-tailed t test. Thereby, the F-test did not calculate any differences between the variances of the individual groups in relation to each other. The one-way ANOVA with Tukey post hoc test was used to calculate: (i) Effect of cisplatin on *RanBP17* gene expression, (ii) differences in the relative content of total RNA or protein per cell and, (iii) relative *RanBP17* gene expression and percentage of cells during different cell cycle phases. For calculating differences of AG1478 treatment on RanBP17 or PCNA protein expression at different stages of the cell cycle, a two-tailed, one-sample t-test was used as the levels of AG1478 treated cells (UM-SSC-3 only) were compared with the expression levels in the corresponding DMSO controls (always set as 1). The GraphPad Prism 4.00 software (GraphPad Software, San Diego, CA) was used for statistical analysis. In all analyses a p value < 0.05 was considered as a significant difference between two groups.

## Results

### Expression of RanBP17 in cells and tissues

Western blot analysis was performed to evaluate RanBP17 protein expression levels in HNSCC tissues (Supplementary Fig. S2A, Supplementary Table S1) and cell lines (Supplementary Fig. S2B, Supplementary Table S2). Most of the tissues and cell lines showed an immunoreactive band consistent with the predicted size (~ 124 kDa) of the RanBP17 reference protein (Q9H2T7-1) but, particularly in cell lines, also other immunoreactive bands of different lower molecular weight. Expression was particularly seen in epithelial cells but also in the two neuroblastoma derived cell lines SKNSH and IMR32. The *RanBP17* gene exhibits a large number of validated and predicted splice variants, with the two protein isoforms Q9H2T7-1 (= RanBP17 reference protein) and Q9H2T7-2 currently being the best characterized ones. The additional lower molecular weight bands as seen during Western blot analysis therefore could represent other isoforms or degradation products of RanBP17. Analysis of cDNA micro array data derived from a previous study [6] demonstrate relative differences of *RanBP17* exon expression levels between the tested HNSCC cell lines, which further supports the notion of different HNSCC cell lines expressing different sets of *RanBP17* splice variants (Supplementary Fig. S3).

### **RNAi knockdown of*RanBP17*inhibits cellular proliferation independent of CDDP**.

**Fig. 1:**
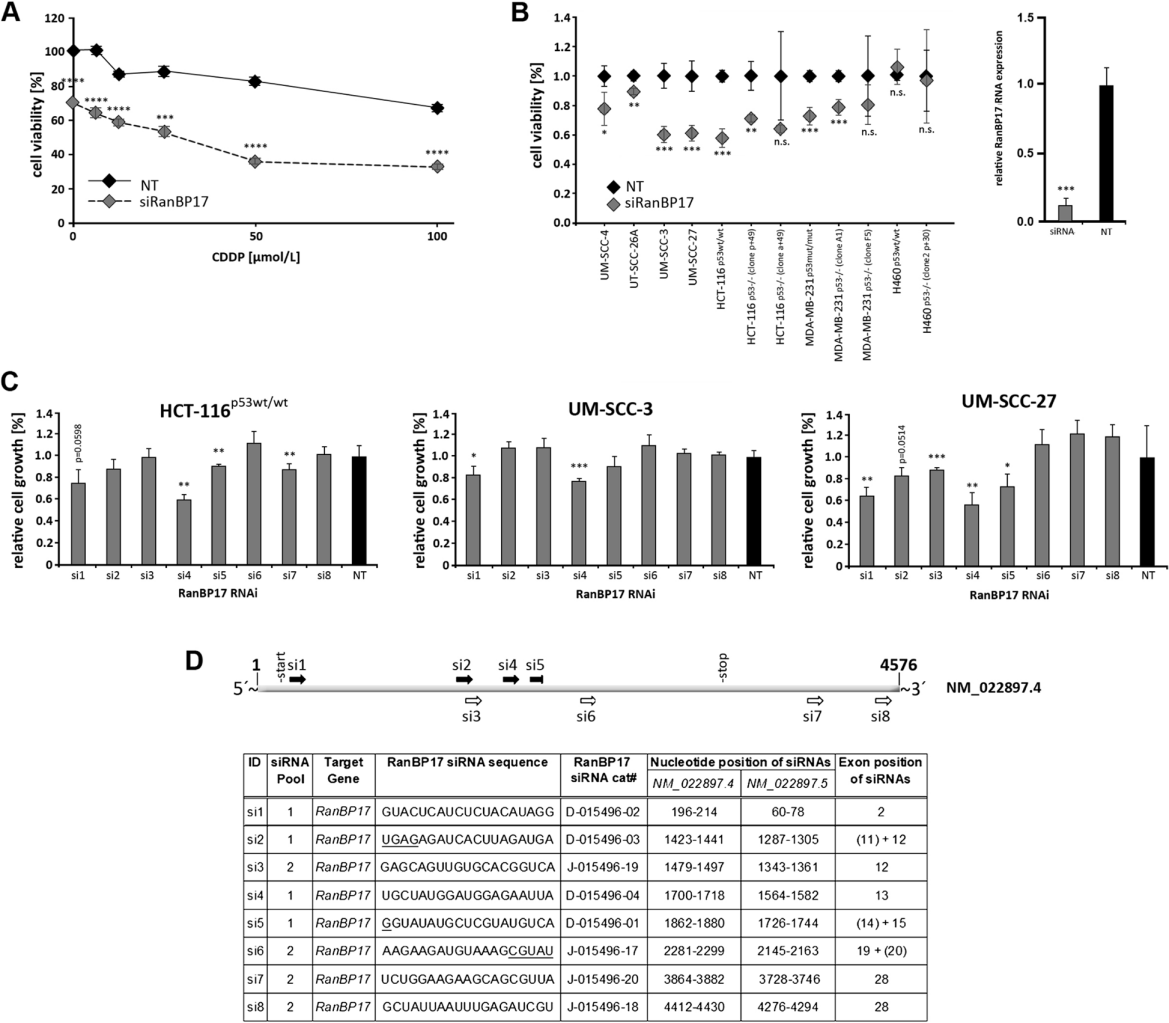
RNAi knockdown of *RanBP17* inhibits cell proliferation independent of CDDP treatment. **A** XTT proliferation assay depicting the relative cell viability of the HNSCC cell line UM-SCC-3 after RNAi knockdown of *RanBP17* using pool 1 siRNA (consists of si1, si2, si4, si5 as depicted in **D**) following incubation with CDDP (6.25, 12.5, 25, 50 and 100 µmol/L). Note the significant reduction in cell viability at 0 µmol/L CDDP in UM-SCC-3 cells after *RanBP17* knockdown. **B** Effect of *RanBP17* RNAi knockdown alone (using pool 1 siRNA) was tested in the indicated cell lines. Efficient RNAi knockdown of *RanBP17* RNA using pool 1 siRNA is shown for HCT116^p53 wt/wt^ cells. **C** The 3 most responsive cell lines from **B** were used to evaluate the effect of all 8 single *RanBP17* specific siRNAs (si1-8). **D** Depicted are the siRNA (si1-8) binding sites as well as their location within the *RanBP17* reference sequence NM_022897.4. Also included is the respective information for the updated *RanBP17* reference sequence NM_022897.5, which differs from NM_022897.4 by lacking the first 136 nucleotides. Underlined nucleotides refer to the nucleotide sequence of the adjacent exon (shown in brackets). NT_non target RNA, siRanBP17=silencing RNA (pool 1) directed against RanBP17. Statistical tests; A, B, C: Unpaired Students t-test, two-sided (*n*=4 for all analyses) *p<0.05, **p<0.01, ***p<0.001, ****p<0.0001

Since *RanBP17* initially was found differentially expressed in CDDP resistant and sensitive HNSCC cell lines, it was interesting to evaluate if RanBP17 is implicated in CDDP resistance of these cells. For this, RNAi knockdown of *RanBP17* using a pool of 4 *RanBP17* specific siRNAs (si1, si2, si4 and si5 (= Pool 1); Fig. 1) was performed in the HNSCC cell line UM-SCC-3, which subsequently was treated with rising levels of CDDP. Surprisingly, even without addition (0 µmol/L) of CDDP, HNSCC cells exhibited a dramatic reduction in cellular proliferation as seen in the XTT viability assay (Fig. 1A). This observation pointed to RanBP17 possibly being involved in cell proliferation.

### ***RanBP17*RNAi knockdown - associated inhibition of proliferation is a general phenomenon in cell lines**

Subsequently, *RanBP17* RNAi knockdown was performed on a panel of HNSCC cell lines (UM-SCC-4, UT-SCC-26A, UM-SCC-3 and UM-SCC-27) including a colon (HCT116), a lung (H460) and a breast (MDA-MB-231) cancer cell line, the latter three together with their respective CRISPR/Cas9 *TP53* knockout counterparts (Fig. 1B). A significant growth inhibitory effect after *RanBP17* RNAi knockdown (siRNA Pool 1) was seen in most of the tested cell lines (Fig. 1B). Single siRNA analysis of 8 *RanBP17* specific siRNAs (Fig. 1C) derived from pool 1 and pool 2 (si3, si6, si7, si8) (Fig. 1D) revealed the effect of *RanBP1*7 RNAi knockdown on cell proliferation to be associated with specific siRNAs particularly si4, si1 and si5 (Fig. 1C).

### Course of RanBP17 expression in keratinocytes and tumor cell lines after exposure to CDDP

**Fig. 2:**
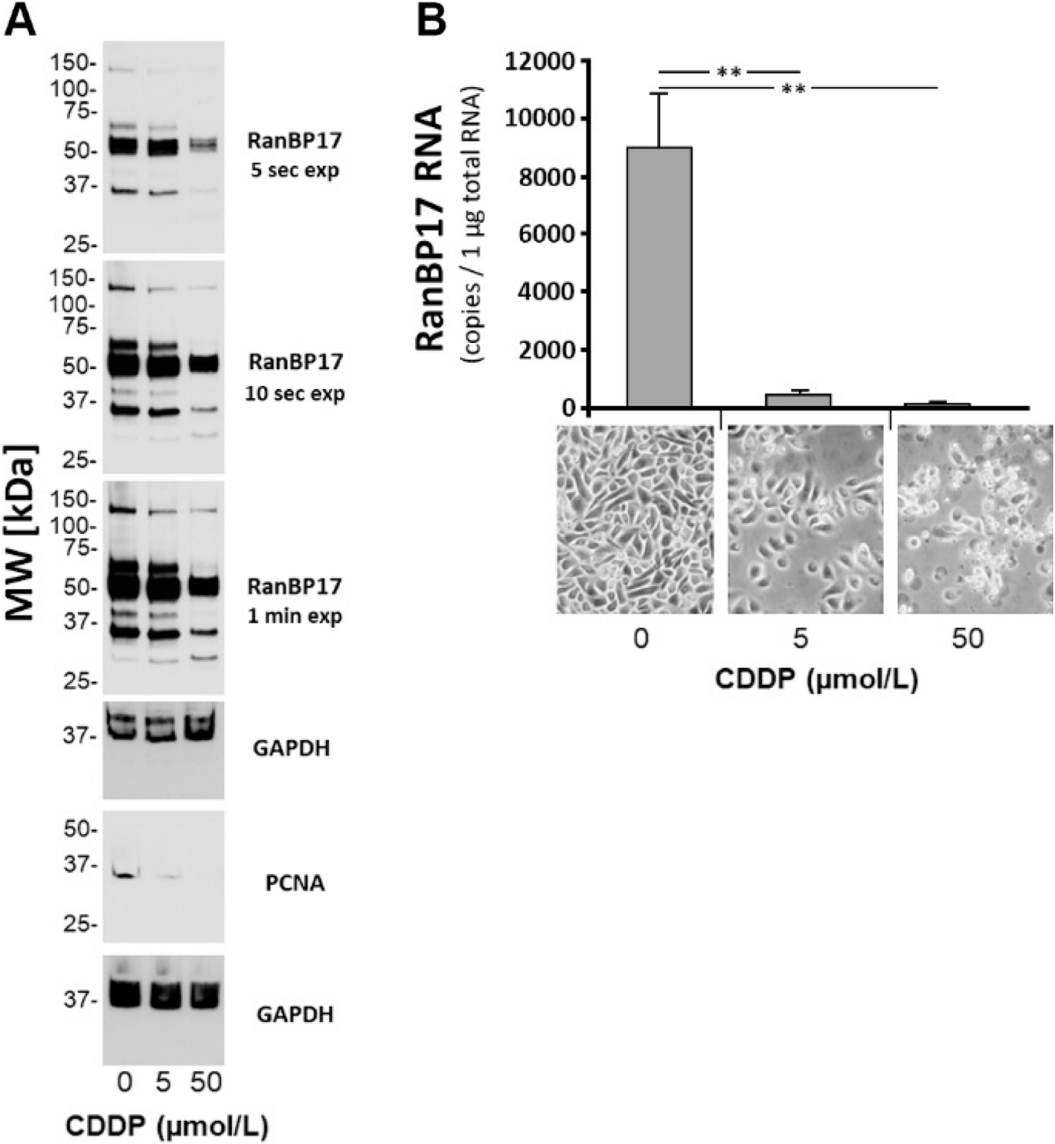
CDDP treatment of normal human epidermal keratinocytes (NHEK) inhibits RanBP17 expression*.* **A** In one preliminary Western Blot experiment, a reduction of RanBP17 protein expression was observed in NHEK, treated with 5 and 50 µmol/L CDDP. **B** RT-qPCR analysis was subsequently performed to evaluate the response of *RanBP17* RNA expression levels on CDDP treatment. Statistical tests in **B** One way ANOVA with Tukey´s correction for multiple comparisons (*n*=4) **p<0.01

**Fig. 3:**
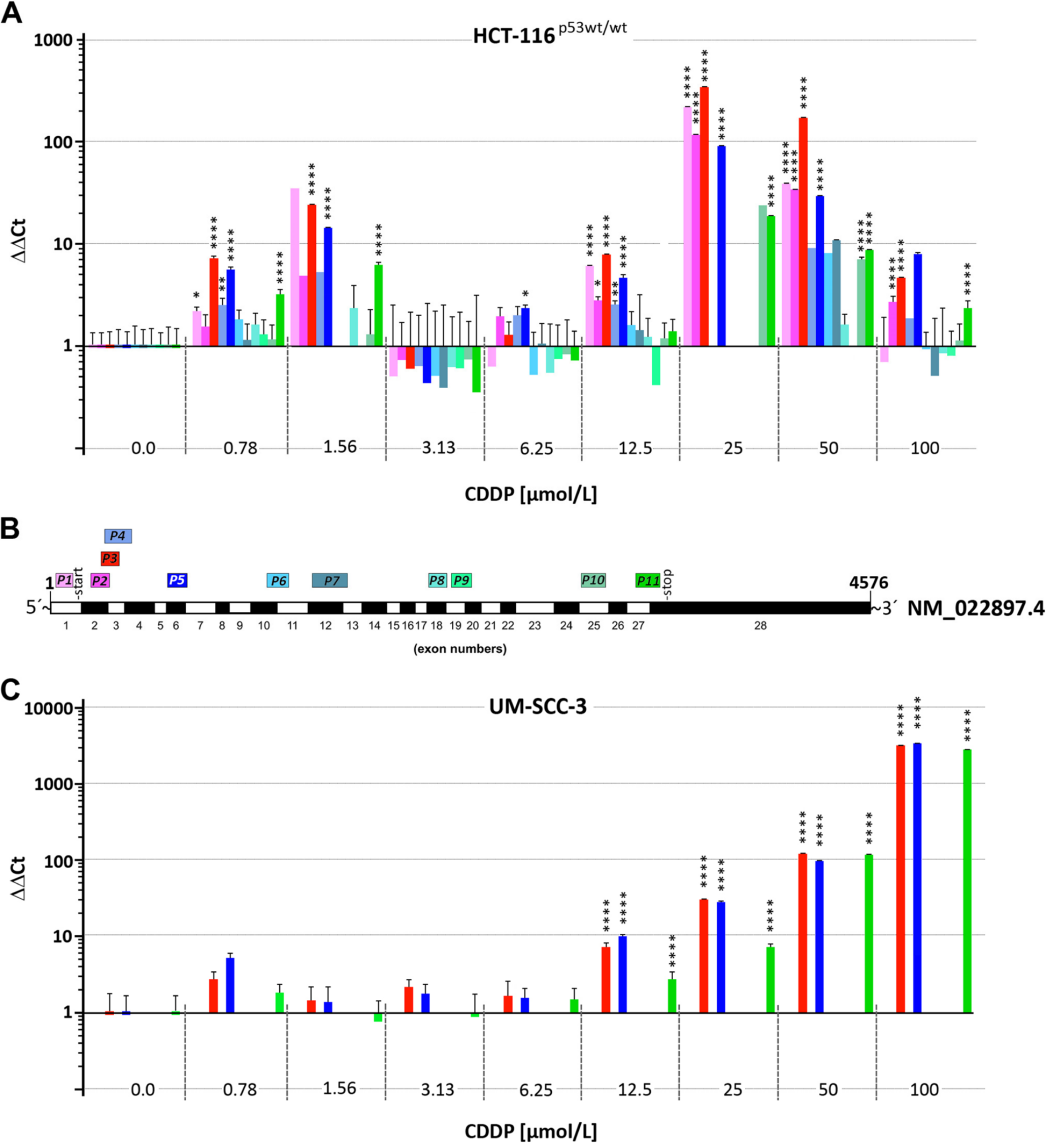
Course of *RanBP17* expression in HCT116^p53wt/wt^ and UM-SCC-3 tumor cells after CDDP treatment. **A ***RanBP17* RNA expression at different CDDP concentrations (0, 0.78, 1.56, 3.13, 6.25, 12.5, 25, 50, 100 µmol/L) is shown for HCT116^p53wt/wt^ cells using 11 different primer pairs (**B**). The same is shown for the UM-SCC-3 cell line (**C**). Here only 3 primer pairs (P3, P5, P11) amplified PCR products. Background information for primer pairs P1-P11 are depicted in Supplementary Fig. S4. Statistical tests: One way ANOVA with Tukey´s correction for multiple comparisons (*n*=1-6) *p<0.05; **p<0.01; ****p<0.0001

At this point, the data indicated RanBP17 to be required for cell proliferation. Contrariwise, to evaluate if inhibiting cellular proliferation affects RanBP17 expression levels, NHEK were treated with CDDP. RanBP17 expression levels were monitored by Western blot (Fig. 2A) and RT-qPCR analysis (Fig. 2B). At 5 and 50 µmol/L CDDP, expression of RanBP17 dropped at the protein (Fig. 2A) and RNA (Fig. 2B) level thereby further implicating RanBP17 with cellular proliferation. Furthermore, *RanBP1*7 RNA expression levels were monitored by RT-qPCR in HCT116^p53 wt/wt^ and UM-SCC-3 tumor cell lines after treatment with 0.78, 1.56, 3.13, 6.25, 12.5, 25, 50 and 100 µmol/L CDDP using eleven (P1-P11) *RanBP17* specific primer pairs (Fig. 3, Supplementary Fig. S4). Here, *RanBP17* RNA levels at most of the tested CDDP concentrations appeared induced. However, particularly pronounced in HCT116^p53wt/wt^ cells at 3.13 µmol/L CDDP, *RanBP17* RNA levels appeared reduced compared with basal levels (Fig. 3A). Interestingly, in UM-SCC-3 cells, only primer pairs P3, P5 and P11 yielded respective *RanBP17* amplicons whereas all primer pairs (P1-P11) worked for HCT116^p53 wt/wt^ cells (Fig. 3C, Supplementary Fig. S4).

### RanBP17 expression levels during cell cycle

**Fig. 4:**
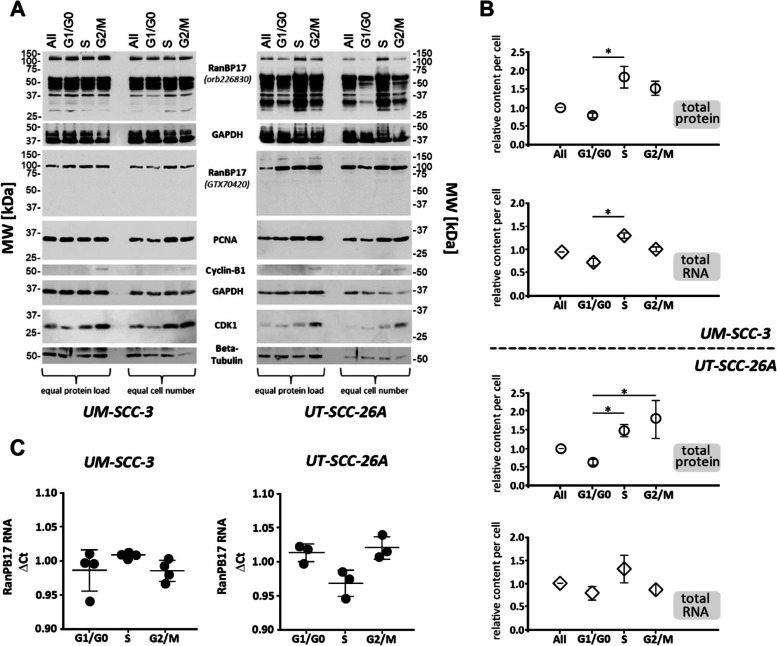
Expression of RanBP17 during the cell cycle*.* **A** FACS sorting yields 4 cell populations, representing the G1/G0, S and G2/M phases of the cell cycle as well as a population labeled „All“, which contains all phases for comparison. Western blot analysis points to increased expression levels of RanBP17 (antibody orb226830) during the S-phase of the cell cycle, which is particularly prominent in UT-SCC-26A. Cell cycle associated proteins (PCNA, cyclin-B1, CDK1) show an expected increase of their expression during S- and into G2/M-phase, thereby helping to validate the accuracy of cell sorting to the respective phase of the cell cycle. **B** Total cellular protein was significantly elevated in S phase cells compared with cells in the G1/G0 phase of the cell cycle, which is an expected finding for cells in division. A similar observation was made for total RNA levels. **C** No significant differences in *RanBP17* RNA levels could be noted between the different cell cycle phases for UM-SCC-3 (*n*=4) and UT-SCC-26A (*n*=3) cells. Statistical tests: B and C: One way ANOVA with Dunn´s correction for multiple comparisons. *p<0.05. (A: Protein bands shown in the upper two panels (RanBP17-orb226830 and GAPDH) and the lower six panels (RanBP17-GTX70420, PCNA, Cyclin-B1, GAPDH, CDK1, Beta-Tubulin) are derived from lysates of two independent experiments). *TP53* mutation in UM-SCC-3: p.R248Q (nuclear p53); *TP53* mutation in UT-SCC-26A: p.Y236* (cytoplasmic p53)

To further clear up the role of RanBP17 in cell proliferation, two HNSCC cell lines, UM-SCC-3 and UT-SCC-26A, were sorted according to their respective cell cycle phases (G1/G0, S, G2/M) and RanBP17 RNA and protein expression levels were evaluated for cells from each cell cycle phase (Fig. 4). Cell cycle phase associated proteins (PCNA, CDK1, cyclin-B1) were carried along to validate successful sorting of cell cycle phase cell populations (Fig. 4A). Shown in Fig. 4B is the “relative protein content per cell” by measuring the total protein concentration for each of the 4 samples (All, G1/G0, S, G2/M) and adjusting it to the respective cell number. Here, S-phase cells expressed more total protein (and total RNA) per cell than G1/G0 cells, as would be anticipated from cells in this phase of the cell cycle thereby further confirming a successful sorting of cells according to their cell cycle phase. S-phase cells appeared to exhibit elevated RanBP17 protein levels especially when compared with G1/G0-phase cells (Fig. 4A) but this observation could not be unequivocally validated in our study. Similarly, no significant differences in *RanBP17* RNA expression levels could be seen between cells of different cell cycle phases (Fig. 4C). Interestingly, in Fig. 4A, an alternative RanBP17 antibody (GTX70420) was used in addition to the regularly deployed RanBP17 antibody (orb226830). The difference between both antibodies is that one binds to an N-terminal (orb226830) and the other to a C-terminal (GTX70420) epitope of the RanBP17 reference protein. Only the N-terminal antibody also detected major additional RanBP17 specific bands between 40 and 70 kDa, whereas the C-terminal antibody detected only one major band. The smaller RanBP17 bands therefore appear to contain N-terminal but not C-terminal epitopes of RanBP17, which is consistent with the presence of splice isoforms or C-terminally degraded RanBP17 proteins. It is noteworthy, that the C-terminal antibody appears to recognize a slightly smaller RanBP17 protein than expected for the respective reference protein.

### AG1478-induced G1-arrest results in accumulation of RanBP17

**Fig. 5:**
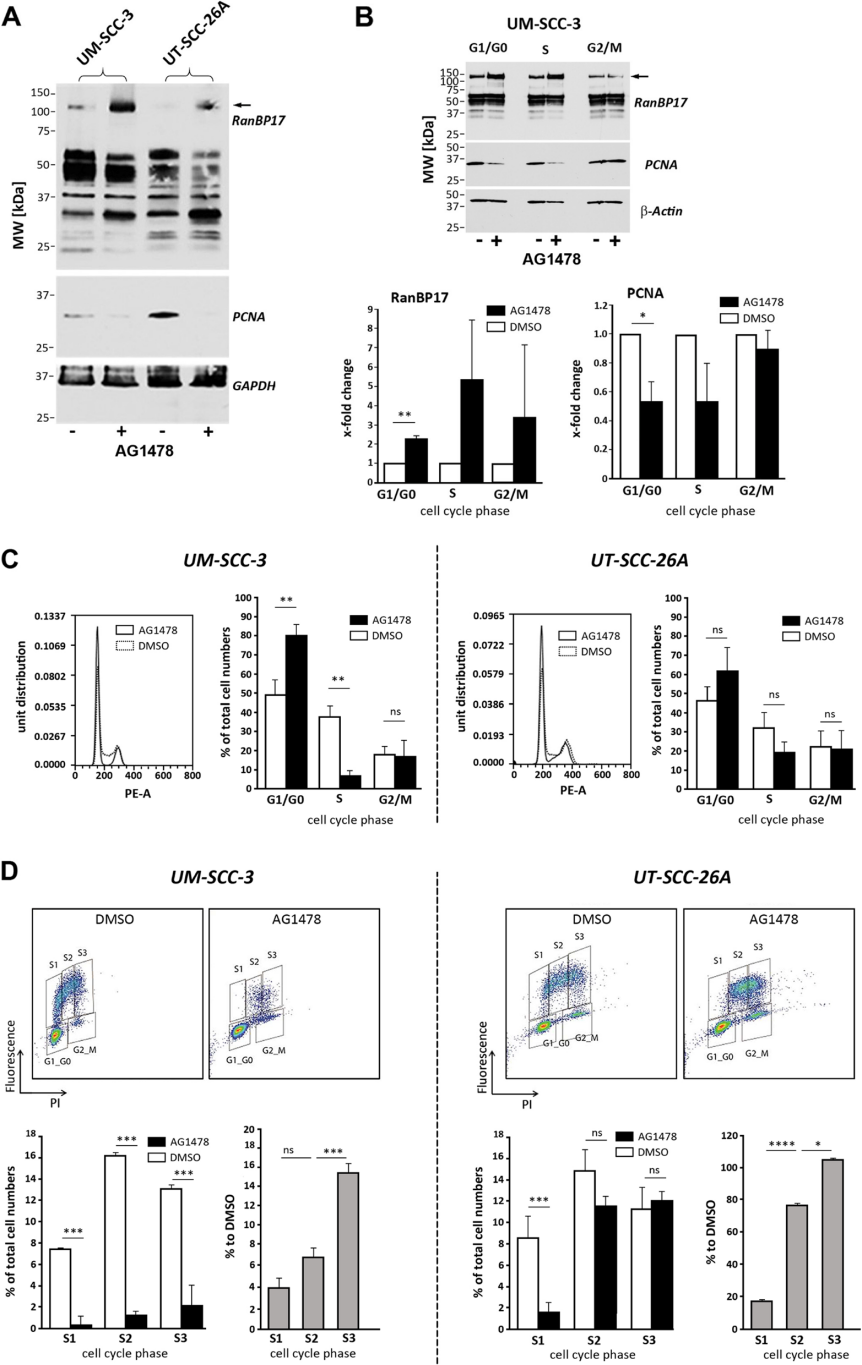
Treatment with the EGFR kinase inhibitor AG1478 arrests cells in G1 and leads to accumulation of RanBP17*.* **A** Treatment of UM-SCC-3 and UT-SCC-26A HNSCC cell lines with the EGFR kinase inhibitor AG1478 reduced cell proliferation (PCNA) but displayed an unexpected rise of RanBP17 protein expression levels (arrow, antibody orb226830). **B** Cell cycle sorting of UM-SCC-3 cells reveals a significant rise of RanBP17 and drop of PCNA in G1/G0 cells after AG1478 treatment. **C** Cell cycle analysis shows a reduction of cells in the S and elevation of cells in the G1/G0 phase of the cell cycle after AG1478 exposure (significant in UM-SCC-3) consistent with a block in the G1 phase as previously reported for this kinase inhibitor [21]. **D** A FACS based BrdU proliferation assay was performed for more detailed S-phase analysis. The S-phase was separated into three subcompartments (S1-S3, gates were adjusted manually for each sample to account for shifts in the whole cell population). A significant drop of cells in the early S phase (S1) was observed in both tested HNSCC cell lines. Cell numbers in S2 and S3 were significantly reduced in UM-SCC-3 but not UT-SCC-26A cells. Statistical tests: **C** (column chart) and **D** (left column chart): Unpaired Students t-test, two-sided; **D** (right column chart): One way ANOVA with Tukey´s correction for multiple comparisons. (*n*=4 for all analyses) *p<0.05, **p<0.01, ***p<0.001, ****p<0.0001. *TP53* mutation in UM-SCC-3: p.R248Q (nuclear p53); *TP53* mutation in UT-SCC-26A: p.Y236* (cytoplasmic p53)

Proliferation and progression of HNSCC tumors is highly dependent on the activity of the receptor tyrosine kinase EGFR (epidermal growth factor receptor) [20]. We deployed the small molecule EGFR kinase inhibitor AG1478 to evaluate if inhibition of EGFR mediated HNSCC cell proliferation also affects RanBP17 expression levels. Unexpectedly, RanBP17 protein expression was markedly induced after AG1478 treatment in the two tested HNSCC cell lines UM-SCC-3 and UT-SCC-26A, with a concomitant expected reduction of PCNA levels (Fig. 5A). Cell cycle sorting of the UM-SCC-3 cell line, which showed the most pronounced induction of RanBP17, revealed a significant rise of RanBP17 and reduction of PCNA protein levels in cells of the G1/G0 population after kinase inhibitor treatment (Fig. 5B). FACS analysis demonstrated signs of an AG1478 induced G1 arrest in both cell lines, as reported earlier [21], with a rise of the G1 cell population and drop of S phase cell numbers, which reached significance in UM-SCC-3 cells (Fig. 5C). Interestingly, deploying the BrdU assay revealed cells in the early S-phase to be particularly affected by AG1478 (Fig. 5D).

### Subcellular localization of RanBP17

**Fig. 6:**
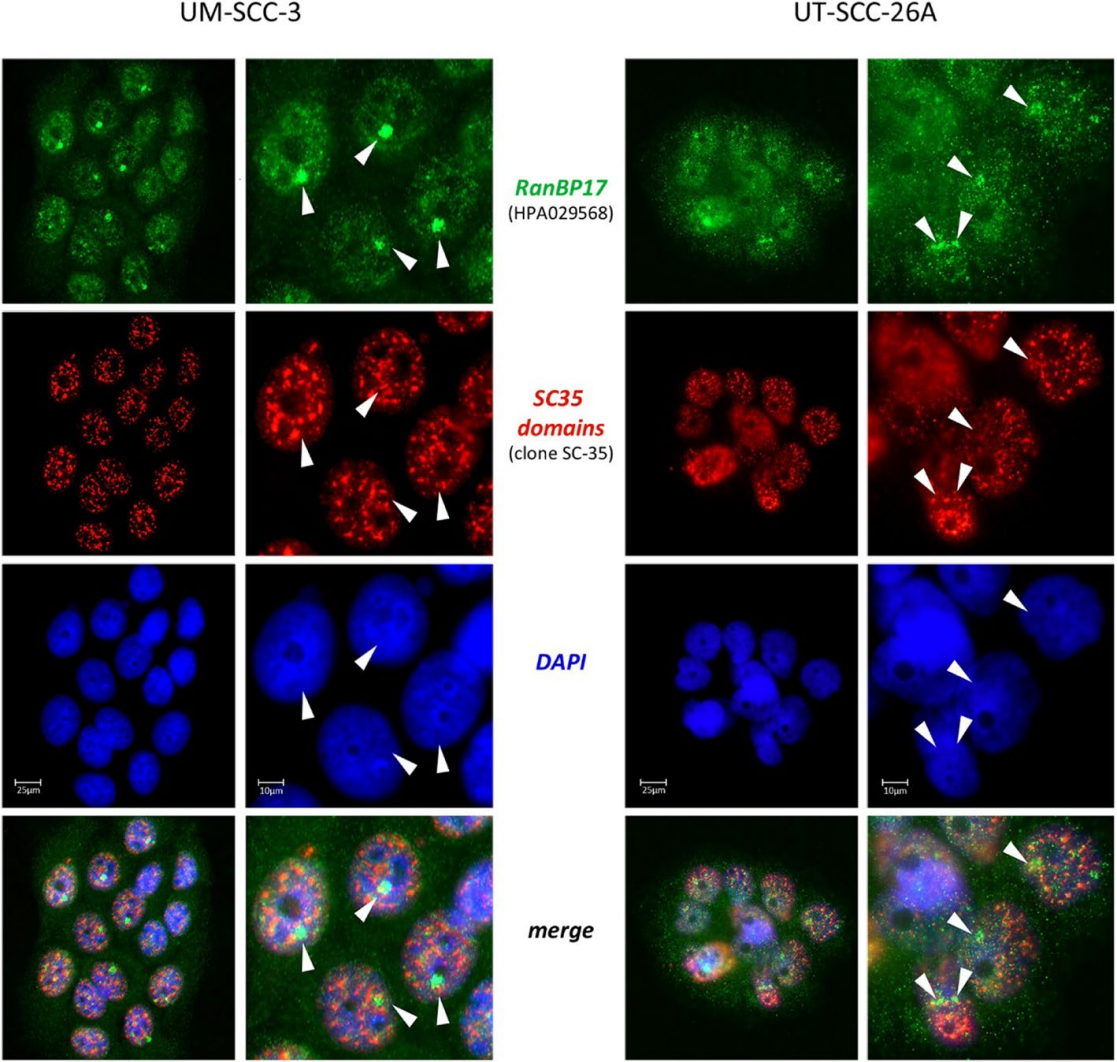
RanBP17 localizes to nuclear bodies distinct from SC35 domains. UM-SCC-3 and UT-SCC-26A cells were co-stained with the RanBP17-specific antibody HPA029568 and a SC35-specific antibody (clone SC-35), exhibiting a distinct nuclear localization of RanBP17 (arrowheads) that is different from SC35 domains. DAPI was used to counterstain the nucleus. *TP53* mutation in UM-SCC-3: p.R248Q (nuclear p53); *TP53* mutation in UT-SCC-26A: p.Y236* (cytoplasmic p53)

**Fig. 7:**
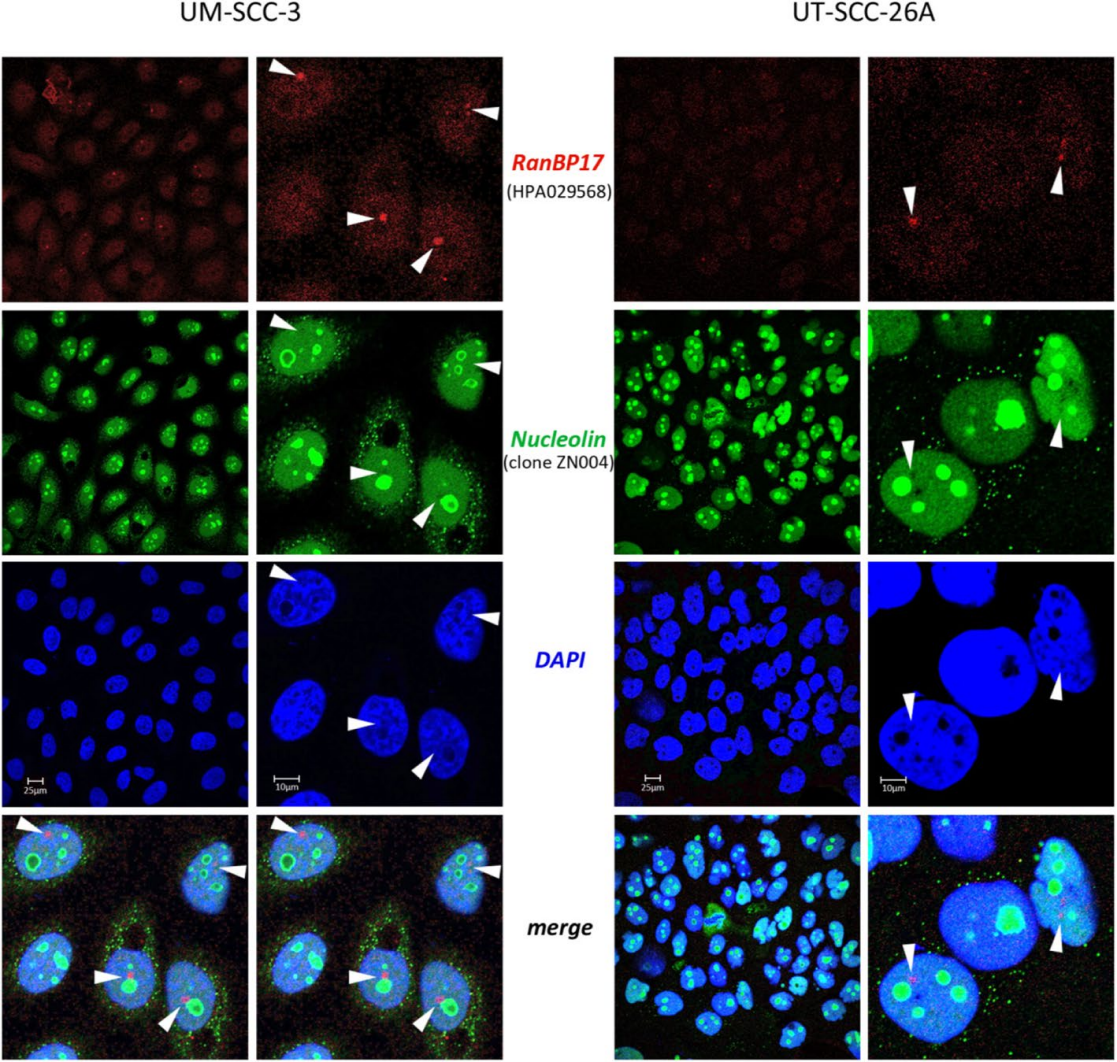
RanBP17 does not localize to nucleoli. UM-SCC-3 and UT-SCC-26A cells were co-stained with the RanBP17-specific antibody HPA029568 and an antibody directed against Nucleolin (clone ZN004), exhibiting a distinct nuclear RanBP17 localization (arrowheads) that is different from nucleoli. DAPI was used to counterstain the nucleus. *TP53* mutation in UM-SCC-3: p.R248Q (nuclear p53); *TP53* mutation in UT-SCC-26A: p.Y236* (cytoplasmic p53)

Previous reports demonstrated colocalization of RanBP17 with nuclear bodies particularly so-called nuclear speckles [9], also known as interchromatin granule clusters or SC35 domains, which are the places of pre-mRNA splicing [22]. In addition to these previous reports, when using a highly validated RanBP17 antibody directed against the central portion (aa 518–593) of the RanBP17 reference protein (Q9H2T7-1), we could not see a major colocalization with SC35 domains (Fig. 6). Specificity of the RanBP17 signal was confirmed by loss of RanBP17 fluorescence after preabsorbing the RanBP17 antibody with its corresponding peptide antigen (Supplementary Fig. S5). The observed phenotype corresponds to RanBP17 positive nuclear bodies distinct from SC35 domains or nucleoli (Figs. 6 and 7) and is consistent with data from The Human protein Atlas (https://www.proteinatlas.org/ENSG00000204764-RANBP17/cell). Although not colocalizing with nucleoli, the RanBP17 positive bodies or clusters on occasion were found adjacent to nucleoli (Fig. 7). These RanBP17 positive nuclear bodies appeared in low numbers and were inconsistent and only present in a subset of cells. According to previous reports these nuclear bodies, marked by RanBP17, resemble so-called clastosomes, which are nuclear bodies rich in proteasomes [23]. However, we could not yet demonstrate that these nuclear RanBP17 clusters clearly correspond to clastosomes.

### Association of *RanBP17* with improved survival in patients with HPV+ HNSCC

**Fig. 8:**
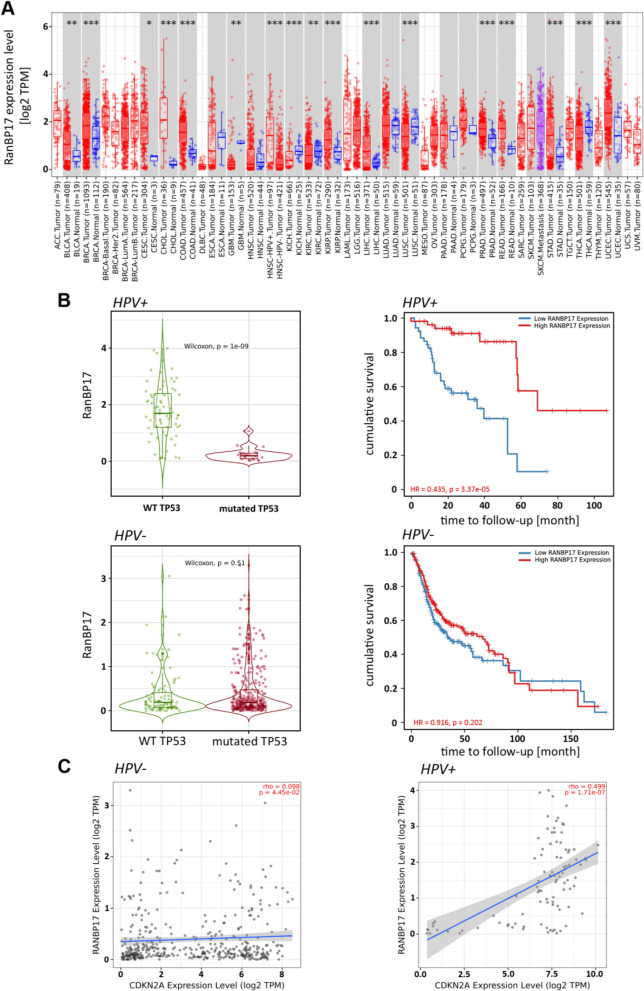
Timer2.0 analysis (https://timer.cistrome.org/) showing **A** the Gene_DE comparison of the Exploration tab for the different TCGA entities for *RANBP17* and **B** the comparison for HPV+ (upper part) and HPV- (lower part) samples within the TCGA-HNSC (HNSCC, head and neck squamous cell carcinoma) cohort regarding *RANBP17* expression and *TP53* mutational status with correlating age adjusted Kaplan-Meier (cumulative survival) plot based on a 50% *RanBP17* expression split (accessible at Gene_Outcome and Gene Mutation of the Exploration tab). **C** comparison of *RanBP17* and *CDKN2A* (p16^INK4A^). *: *p*-value < 0.05; **: *p*-value <0.01; ***: *p*-value <0.001 (Wilcoxon test). HPV-: human papilloma virus negative; HPV+: human papilloma virus positive; Abbreviations according to The Cancer Genome Atlas (TCGA). TPM: transcripts per kilobase million

The possible influence of *RanBP17* expression on HNSCC and other malignancies was evaluated using the TCGA database. Comparing tumor tissues with normal controls frequently revealed significantly different *RanBP17* expression levels between the tested tissues. In particular, HPV+ HNSCC showed significantly higher *RanBP17* expression levels compared with HPV- HNSCC (Fig. 8A). When comparing the survival of HPV+ HNSCC patients carrying *RanBP17* high or low (cutoff 50%) expressing HNSCC, a significantly higher cumulative survival is seen for patients with *RanBP17* high HPV+ HNSCC. However, no such difference was observed in patients with HPV- HNSCC (Fig. 8B, Kaplan -Meier plots). Interestingly, higher *RanBP17* expression levels were also associated with the presence of wild-type *TP53* but only in HPV+ HNSCC (Fig. 8B, violin plots). Furthermore, a highly significant correlation was seen between *RanBP17* and *CDKN2A*, the gene encoding the cell cycle inhibitor and  HPV surrogate marker p16^INK4A^. Since HPV+ HNSCC are frequently associated with high levels of tumor infiltrating leukocytes (TILs) [24], the observed *RanBP17* overexpression could also be derived from TILs present in the tumor. To further evaluate this aspect, we first looked into T-cell and B-cell marker expression levels and, consistent with previous reports, could observe significantly higher levels of these markers in HPV+ HNSCC compared with HPV- HNSCC (Supplementary Fig. S6A). Similarly, the best correlation of *RanBP17* with the bona fide immune marker candidates CD8A, CD4 and CD274 was seen in HPV+ HNSCC (Supplementary Fig. S6B). To evaluate the *RanBP17* expression levels of different cell types present in the tumor tissue, we used existing single cell RNAseq (scRNAseq) data sets [25, 26]. As depicted in Supplementary Fig. S7A, immune cells exhibit only low *RanBP17* expression levels and therefore do not appear to account for the elevated *RanBP17* levels in tumor tissues with high TIL levels as found in HPV+ HNSCC. However, this dataset [25] only considered HPV- HNSCC. To evaluate, if HPV positivity of a tumor could affect *RanBP17* expression in TILs, we looked into another dataset, which included scRNAseq data from HPV- and HPV+ HNSCC derived immune cells [26]. Here, no difference in *RanBP17* levels could be detected between immune cells derived from HPV- and HPV+ HNSCC (Supplementary Fig. S7B).

### Possible implications of circular *RanBP17* RNA in HNSCC

In a recent study by Zhou and coworkers, the authors identified a *RanBP17* derived circular RNA (*circRanBP17*) that is implicated in the pathogenesis of nasopharyngeal cancer [27]. The authors demonstrated *circRanBP17* to promote nasopharyngeal cancer cell proliferation and invasion by acting via mir-635 and RUNX2 and pointed out *circRanBP17* as a potential target for nasopharyngeal cancer treatment. This report further highlights a role of RanBP17 and its derivatives in tumor progression. To address the miR-635/RUNX2 axis, we additionally compared the expression of *RUNX2*, in the presence of wild-type or mutant *TP53*, in the HPV- (Supplementary Fig. S8A, left) and HPV+ (Supplementary Fig. S8B, left) HNSCC groups. Here, no significant differences in *RUNX2* expression was seen between the wild-type or mutant *TP53* groups whether in the HPV- nor in the HPV+ HNSCC group. In addition, we performed correlation analyses of *RUNX2* and *RANBP17* expression for the HPV+ and HPV- groups. Here, a significant correlation was found in the HPV- but not the HPV+ group (Supplementary Fig. S8A and S8B, right).

## Discussion

### Age dependent decrease of RanBP17 levels

Interestingly, in a study by Mertens et al. the authors compared transcriptomes of differently aged human fibroblasts and observed an age dependent decrease of *RanBP17* levels. Similarly, directly induced neurons maintained their aging level, whereas induced pluripotent stem cells (iPSCs) and iPSC-derived neurons did not show age-related features and *RanBP17* reduction. The authors concluded that aging of cells is related to a disturbance of the nucleocytoplasmic transport associated with RanBP17 [28]. The observations of our study are in accordance with the study by Mertens et al. since our data strongly point to an association of RanBP17 with cell proliferation. Age related reduction of cellular RanBP17 levels as reported by Mertens et al. therefore could correlate with a reduced proliferative and regenerative capability of the respective cell. In another study by Kim et al., the authors suggested that RanBP17 could have a critical role for the nucleocytoplasmic transport in uterine endometrium cells during pregnancy [29]. Since pregnancy is highly associated with cell proliferation, these observations further support the assumption that RanBP17 is highly involved in cellular proliferation.

### RanBP17 expression in glioblastoma multiforme

Wang and colleagues compared gene expression patterns between glioblastoma multiforme (GBM) and low grade glioblastoma (LGG) using the TCGA (The Cancer Genome Atlas) database. In their study they could identify a five gene system which allows to predict survival of GBM patients. Among these five genes they also included *RanBP17*, which was the only of the five genes that exhibited a significantly reduced hazard ratio for GBM patients [30]. This finding is in agreement with our observations. Here, in HPV+ HNSCC patients, higher *RanBP17* levels correlated with an improved outcome. It will be interesting to clear up how *RanBP17* is related to a favorable outcome of GBM and HNSCC patients.

### Presence of different RanBP17 isoforms

In the context of seemingly contradictory results regarding RanBP17, it is of utmost importance to again point out that *RanBP17* is a heavily spliced gene, which is giving rise to at least 22 splice variants (Ensembl-Gene: RANBP17 ENSG00000204764). Each of these variants could have a more or less distinct biological function that needs to be further characterized. The existence of different *RanBP17* splice variants was further supported by micro array data of this study showing highly variable hybridization (expression) levels of *RanBP17* exon-specific probes between the tested HNSCC cell lines (Supplementary Fig. S3). Furthermore, in our Western blot analyses, using an antibody directed against the N-terminal region of RanBP17, we observed additional RanBP17 specific bands of smaller size (between 40 and 70 kDa) than expected for the RanBP17 reference protein (~ 124 kDa), that are consistent with the presence of other RanBP17 isoforms. However, these bands could also be the result of a C-terminal degradation of the full size RanBP17 protein. Another RanBP17-specific antibody, directed against the C-terminal region of the protein did not show these smaller sized (app. 40–70 kDa) RanBP17 bands thereby supporting the presence of C-terminally truncated RanBP17 isoforms or degradation products. However, this antibody recognized a single RanBP17 specific band, slightly smaller than expected for the reference protein. Furthermore, it was conspicuous that tumor tissues virtually did not show these additional small bands as seen in many cell lines. Here, we hypothesize that, in contrast to tissues (including tumor tissues) cultured cell lines exhibit a markedly increased cell proliferation and cell turnover rate, which either requires expression of additional RanBP17 splice variants or leads to increased degradation of RanBP17. It will be interesting to further evaluate the potential role of these additional RanBP17 isoforms. Since most studies regarding *RanBP17* do not distinguish between different *RanBP17* splice variants, it remains unclear to which specific RanBP17 isoform or isoforms a respective biological effect can be attributed.

### Possible cargos for RanBP17 mediated transport

Deploying a yeast two-hybrid binding assay, Lee et al. could demonstrate binding of RanBP17 to the basic helix-loop-helix transcription factor E12 [9]. E12 and E47 are alternatively spliced variants of the E2A gene and are particularly known for having a role in lymphocyte development [31]. In their study, the authors proposed that E12 could serve as a cargo for RanBP17 since overexpression of RanBP17 resulted in higher transcriptional activity of E12 and upregulation of p21^Cip1/Waf1^, one of its major targets, thereby implicating RanBP17 to act as an importin rather than an exportin [9]. The role of RanBP17 as an importin is further supported by a study from König et al. in which the authors proposed involvement of RanBP17 in the nuclear import of viral RNA [32]. Interestingly, Bao et al. observed interaction of RanBP17 with sperm maturation protein 1 and suggested a role of RanBP17 in spermatogenesis either as an importin or an exportin [33]. The observations in our study point to a major involvement of RanBP17 in cell proliferation. It therefore appears conceivable that RanBP17 is involved in the nucleocytoplasmic transport of factors required for cell cycle regulation. To further look into possible cargo candidates of RanBP17 and to fathom if this karyopherin acts as an importin or exportin will be an interesting task for future studies.

### RanBP17 is highly expressed in mouse embryonic stem cells (mESCs)

In a study by Sangel and coworkers, the authors investigated the expression of karyopherins in mESc. Here they observed high expression of *RanBP17* in mESCs compared with mouse embryonic fibroblasts (MEFs). RNAi knockdown of *RanBP17* resulted in endodermal differentiation. The authors suggested that RanBP17, among other karyopherins, could be involved in the maintenance of mESC pluripotency [34]. However, in a subsequent study, Sangel demonstrated that overexpression of RanBP17 in MEFs could not promote their reprogramming efficiency into mouse induced pluripotent stem cells (miPSC) [35]. Involvement of *RanBP17* in embryonic stem cells further emphasizes its possible role in cell renewal and proliferation.

### RanBP17 expression levels after CDDP and AG1478 treatment

RanBP17 expression was monitored after inhibiting tumor cells with CDDP or with the EGFR kinase inhibitor AG1478. The most prominent effect seen after CDDP or AG1478 treatment was induction of RanBP17 expression. In contrast to tumor cell lines, CDDP treatment of NHEK resulted in reduction of RanBP17 levels. While we cannot fully understand these differences between tumor cells and NHEK, we currently can only speculate about possible explanations. In this context, it is very tempting to implicate recently discovered *circRanBP17* RNA species as products of the *RanBP17* gene, which are implicated in tumor growth [27]. The presence of, typically non-coding, *circRanBP17* RNA species in the tested cells and cell lines could also explain, why in the micro array analysis (Supplementary Fig. S3) *RanBP17* exons appeared variably expressed among different cell lines and even within the same cell line. This also could explain why several primer pairs did not work in UM-SCC-3 cells (Fig. 3, Supplementary Fig. S4). The presence of *circRanBP17* RNA species, which are generated by back splicing of the *RanBP17* pre-mRNA, typically consist of only few selected exons, e.g. exons 2–5 of the *RanBP17* gene [27], and could account for the observed asymmetrical expression of different *RanBP17* exons. These selective *circRanBP17* RNA species would likely not encode for proteins but could distort the expected correlation of *RanBP17* RNA and protein expression levels in the tested cell lines.

### Possible role of RanBP17 in head and neck cancer

In our study, we observed a major role of RanBP17 in HNSCC cell proliferation, which was also noted in cell lines derived from other tumor types. Surprisingly, TCGA analysis revealed higher *RanBP17* expression levels in HPV+ HNSCC compared with HPV- HNSCC. Furthermore, in patients with HPV+ HNSCC, higher *RanBP17* expression was associated with improved cumulative survival and wild-type *TP53* status. How *RanBP17* is involved in the course of disease in HPV+ HNSCC will be an interesting task for future studies. In this context it is interesting to again mention that RanBP17 was implicated in the nucleocytoplasmic transport of viral (HIV-1) RNA [32]. A similar role could be envisaged for HPV+ HNSCC. In addition, it would be meaningful, to confirm the results of the TCGA data base analysis on a larger number of HNSCC tumor samples, e.g. by using immunohistochemistry and quantitative RT-PCR. If positive, RanBP17 could potentially serve as a novel prognostic marker in HNSCC. This aspect is further supported by the observation that in HPV+ HNSCC, *RanBP17* highly correlates with CDKN2A, the gene encoding the HPV surrogate marker p16^INK4A^. The recent discovery of *circRanBP17* RNA and its implication in nasopharyngeal cancer prompted us to look at a possible involvement of *circRanBP17* in the RanBP17 effects observed in our study. Since *circRanBP17* reportedly was controlled via the “miR-635/RUNX2 axis”, we evaluated a possible correlation of *RUNX2* and *RanBP17* by TCGA analysis. Since no correlation of *RUNX2* and *RanBP17* gene expression was seen in the HPV+ HNSCC group, the observed positive prognostic effect in the *RanBP17*^*high*^ patient population of the HPV+ group does not seem to be dependent on the miR-635/RUNX2 axis. However, in this context, it is interesting to note that in the initial micro array analysis, *RanBP17* was found upregulated in CDDP resistant HNSCC cell lines carrying disruptive *TP53* mutations but at the protein level, no major difference in expression was seen between CDDP resistant and sensitive HNSCC cell lines as would be expected from the micro array data. Therefore, at this point, we cannot exclude that *circRanBP17* could explain this discrepancy. More detailed studies are required to better understand the possible role of *circRanBP17* in HNSCC disease.

## Conclusions

Taken together, RanBP17 appears implicated in cell proliferation. As a member of the importin beta superfamily, RanBP17 could act as an importin or exportin, transporting cargoes such as transcription factors between the cytoplasm and nucleus that are implicated in cell cycle control. To investigate, how *RanBP17* is involved in the prognosis of HPV+ HNSCC will be an interesting task for future studies.

However, investigations into RanBP17 are highly complex due to the fact that numerous *RanBP17* splice variants exist, which could be involved in different even opposite biological functions. Therefore, for future functional studies of RanBP17, it is necessary to take into consideration the presence of different RanBP17 splice isoforms at the RNA and protein level and in particular the possible involvement of *circRanBP17* RNA.

## Supplementary information

**Additional file 1:** **Supplementary Table S1.** Shown is the type and origin of the tissues used for Western blot analysis of RanBP17. **Supplementary Table S2.** Background information of cells and cell lines. **Supplementary Figure S1.** Uncropped Western blot images are shown for Supplementary Figure S2A (A), Supplementary Figure S2B (B), Figure 2A (C), Figure 4A (D), Figure 5A (E) and Figure 5B (F). Arrows point to the predicted size of the respective protein. Dashed rectangles refer to the region of the blot used in the final figure. **Supplementary Figure S2.** Western blot analysis, demonstrating protein expression of RanBP17 in tissues, cells and cell lines. **A**, A band at ~124 kDa (arrows) corresponding to the major RanBP17 isoform (UniProtKB/Swiss-Prot: Q9H2T7) was detected in all tested head and neck tumor tissues (HNSCC_1-15, Adeno CA_1-2) including tonsil (N1) but not muscle (N2) tissues (Supplementary Table S1). **B**, Similarly, a ~124 kDa band was also observed in all head and neck tumor derived cell lines and other cell lines derived from different tumors (Supplementary Table S2). Smaller sized immunoreactive bands are seen in most of the tested samples, especially cell lines. GAPDH was used as an input control. Short and long exposures are shown to visualize strong and weak bands. Indicated at the bottom in **B** is the histologic tumor/tissue type from which cells and cell lines were derived from. epi=epithelial, end=endothelial, mes=mesenchymal, neu=neural, mel=melanocytic, lym=lymphocytic. **Supplementary Figure S3.** Expression of *RanBP17* exon-specific micro array probes in different HNSCC cell lines. To allow for a more precise statement about the differing *RanBP17 *expression levels between the tested HNSCC cell lines during micro array analysis, we also were looking at expression of the individual exons. In order to obtain this information from the microarray files, we first searched for the sample IDs given for *RANBP17* on the Human Gene 1.0 ST Affymetrix chip used. Then, the .CEL files obtained from the microarray sequencing were imported in the program R (v. 4.0.4) using the tool oligo (v 1.54.1) and the function "read.celfiles". Afterwards, a background correction was performed using "backgroundCorrect" followed by normalization using "normalize". Finally, the obtained expression data for the previously searched *RANBP17* Probe IDs were written into a table using write.csv2 and the results were analyzed afterwards. **A**, Background corrected normalized intensity of the respective Probe signal for each tested cell line. **B**, Graphical depiction of the exon position for the single *RanBP17* specific probes according to the *RanBP17 * reference sequence NM_022897.4. **Supplementary Figure S4.** Shown on the left are the primer pairs used for the quantitative PCR analysis in Fig. 3 with their corresponding sequence information. **A** and **B** show comparable efficiencies of all primer pairs in **A**) untreated HCT116^p53wt/wt^ cells and **B**) untreated UM-SCC-3 cells. In UM-SCC-3 cells, only primer pairs P3, P5, and P11 yielded amplicons. **Supplementary Figure S5.** Specificity of the RanBP17 antibody. The RanBP17 signal (left panel in **A, B**) disappears (right panel in **A, B**) after preincubating the RanBP17 antibody (HPA029568) with its corresponding antigen peptide sequence (APrEST73986) thereby confirming specificity of the antibody. *TP53* mutation in UM-SCC-3: p.R248Q (nuclear p53); *TP53* mutation in UT-SCC-26A: p.Y236* (cytoplasmic p53). **Supplementary Figure S6.** Timer2.0 (http://timer.cistrome.org/) was used for the analysis of **A**, the immune association for *RANBP17* and CD8+ T-cells (left side) and B-cells (right side) showing the Spearman correlation coefficients. Color filled boxes indicate significantly higher values (p<0.05) and **B**, for the correlation between the *RANBP17* expression to CD8A/CD4/CD274 for HPV+ (upper part) and HPV- (lower part) samples of the TCGA-HNSC cohort (accessible at Gene_Corr of the Exploration tab), with the blue depicting the linear regression and the grey shaded area depicting the confidence interval. TPM: transcripts per kilobase million. **Supplementary Figure S7. A,** Boxplots for a scRNA-seq (single cell RNA-sequencing) HNSCC dataset (GEO103322) showing the expression of *RANBP17*, *CD8a*, *CD4*, and *CD274* for the different cell types. Each dot represents the expression of one sequenced cell. **B**, Violin plots for the scRNA-seq immune cell HNSCC dataset (GEO139324), for which the expression details for each cell were extracted from the provided matrix files using Seurat for R. Plots were generated using the modules matplotlib and seaborn for python. **Supplementary Figure S8.** Boxplot analysis of the *TP53* mutational status with regard to *RUNX2 *expression as well as correlation analysis of *RUNX2 *with *RanBP17* for the **A**, HPV- and **B**, HPV+ TCGA HNSCC cohorts, showing no significant expression differences with regard to *TP53* mutational status but a significant correlation in the HPV- cohort only. Analyses were performed using the TIMER2.0 Gene_Mutation module (http://timer.cistrome.org/). Left graphs: Wilcoxon test. Right graphs: linear regression with Spearman’s rho.

## Data Availability

Data used and/or analyzed during the current study is available from the corresponding author on reasonable request. The results shown in Fig. 8 and Supplementary Figs. S6, S7 and S8 are in whole or part based upon data generated by the TCGA Research Network: https://www.cancer.gov/tcga.

## Abbreviations

ABC: ATP-binding Cassette
ANOVA: Analysis of variance
BSA: Bovine serum albumin
CDDP: Cis-diamminedichloroplatinum (II)
CDK1: Cyclin-dependent kinase 1
cDNA: Complementary DNA
circRanBP17: Circular RanBP17
DMSO: Dimethyl sulfoxide
CRISPR/Cas9: Clustered regularly interspaced short palindromic repeats/CRISPR associated protein 9
FACS: Fluorescence activated cell sorting
FBS: Fetal bovine serum
FCS: Fetal calf serum
GAPDH: Glyceraldehyde 3-phosphate dehydrogenase
GBM: Glioblastoma multiforme
HNSCC: Head and neck squamous cell carcinoma
HPV: Human papilloma virus
HRP: Horseradish peroxidase
HUVEC: Human umbilical vein endothelial cells
IgG: Immunoglobulin G
iPSC: Induced pluripotent stem cells
mESC: Mouse embryonic stem cells
NHEK: Normal human epidermal keratinocytes
NT: Non target
PCNA: Proliferating cell nuclear antigen
RanBP17: Ran GTP binding protein 17
PBS: Phosphate buffered saline
RNAi: RNA interference
RT-qPCR: Reverse transcription quantitative polymerase chain reaction
SC35: Serine/arginine-rich splicing factor
siRNA: silencing RNA
TCGA: The cancer genome atlas
TILs: Tumor infiltrating leukocytes
XTT: 2,3-Bis-(2-methoxy-4-nitro-5-sulfophenyl)-2*H*-tetrazolium-5-carboxanilide
