# Supplementary material for: The importin beta superfamily member RanBP17 exhibits a role in cell proliferation and is associated with improved survival of patients with HPV+ HNSCC
Source: BMC Cancer. 2022 Jul 18;22:785. doi: 10.1186/s12885-022-09854-0 (PMC9290296; doi:10.1186/s12885-022-09854-0)
Supplement: Supplementary file 1 — Additional file 1: Supplementary Table S1. Shown is the type and origin of the tissues used for Western blot analysis of RanBP17. Supplementary Table S2. Background information of cells and cell lines. Supplementary Figure S1. Uncropped Western blot images are shown for Supplementary Figure S2A (A), Supplementary Figure S2B (B), Figure 2A (C), Figure 4A (D), Figure 5A (E) and Figure 5B (F). Arrows point to the predicted size of the respective protein. Dashed rectangles refer to the region of the blot used in the final figure. Supplementary Figure S2. Western blot analysis, demonstrating protein expression of RanBP17 in tissues, cells and cell lines. A, A band at ~124 kDa (arrows) corresponding to the major RanBP17 isoform (UniProtKB/Swiss-Prot: Q9H2T7) was detected in all tested head and neck tumor tissues (HNSCC_1-15, Adeno CA_1-2) including tonsil (N1) but not muscle (N2) tissues (Supplementary Table S1). B, Similarly, a ~124 kDa band was also observed in all head and neck tumor derived cell lines and other cell lines derived from different tumors (Supplementary Table S2). Smaller sized immunoreactive bands are seen in most of the tested samples, especially cell lines. GAPDH was used as an input control. Short and long exposures are shown to visualize strong and weak bands. Indicated at the bottom in B is the histologic tumor/tissue type from which cells and cell lines were derived from. epi=epithelial, end=endothelial, mes=mesenchymal, neu=neural, mel=melanocytic, lym=lymphocytic. Supplementary Figure S3. Expression of RanBP17 exon-specific micro array probes in different HNSCC cell lines. To allow for a more precise statement about the differing RanBP17 expression levels between the tested HNSCC cell lines during micro array analysis, we also were looking at expression of the individual exons. In order to obtain this information from the microarray files, we first searched for the sample IDs given for RANBP17 on the Human Gene 1.0 ST Affymetrix chip used. Then, [file 12885_2022_9854_MOESM1_ESM.pdf]

Supplementary Table S1. Shown is the type and origin of the tissues used for Western blot analysis of RanBP17.

Name	Type of tissue	Anatomic location	Patient age	M/F
**Adeno CA_1**	adenocarcinoma	paranasal sinus	70y	F
**Adeno CA_2**	adenocarcinoma	paranasal sinus	71y	F
**HNSCC_1**	squamous cell carcinoma	tongue	74y	F
**HNSCC_2**	squamous cell carcinoma	larynx (epiglottic)	57y	M
**HNSCC_3**	squamous cell carcinoma	hypopharynx (piriform sinus)	61y	M
**HNSCC_4**	squamous cell carcinoma	larynx	69y	M
**HNSCC_5**	squamous cell carcinoma	larynx	72y	M
**HNSCC_6**	squamous cell carcinoma	larynx	59y	M
**HNSCC_7**	squamous cell carcinoma	larynx	67y	F
**HNSCC_8**	squamous cell carcinoma	larynx	41y	M
**HNSCC_9**	squamous cell carcinoma	floor of mouth	45y	M
**HNSCC_10**	squamous cell carcinoma	hypopharynx	69y	F
**HNSCC_11**	squamous cell carcinoma	larynx (glottic)	68y	M
**HNSCC_12**	squamous cell carcinoma	oropharynx (tonsil)	78y	M
**HNSCC_13**	squamous cell carcinoma	relaps (tracheostoma)	43y	M
**HNSCC_14**	squamous cell carcinoma	larynx	43y	M
**HNSCC_15**	squamous cell carcinoma	oropharynx (tonsil)	56y	F
**N1**	normal	tonsil	-	-
**N2**	normal	muscle	54y	M

HNSCC=head and neck squamous cell carcinoma; M=male; F=female; y=years; “-“= no information

Supplementary Table S2. Background information of cells and cell lines.

Name	Tissue type	Species	Viral status	p53 status	Anatomic location	Patient age	M/F
**293T RRID: CVCL_0063**	Transformed cell line (epi)	HS	SV40 polyomavirus *large T antigen*	WT	Kidney	Fetus	F
**A7 RRID: CVCL_3486**	Melanoma (mel)	HS	-	-	Skin	-	-
**A431 RRID: CVCL_0037**	Epidermoid carcinoma (epi)	HS	-	c.818G>A p.R273H	Skin	85y	F
**COS7 RRID: CVCL_0224**	Transformed cell line (epi)	CA	SV40 polyomavirus *large T antigen*	WT	Kidney	Adult	M
**HaCaT RRID: CVCL_0038**	Skin (epi)	HS	-	c.535C>T p.H179Y c.843_844delinsTT p.R282W	Back	62y	M
**HeLa RRID: CVCL_0030**	Adeno-carcinoma (epi)	HS	HPV-18	WT	Uterus, cervix	30.5y	F
**H460 RRID: CVCL_0459**	Lung large cell carcinoma (epi)	HS	-	WT	Lung	-	M
**HCT-116 RRID: CVCL_0291**	Carcinoma (epi)	HS	-	WT	Colon	48y	M
**HT29 RRID: CVCL_0320**	Adeno-carcinoma (epi)	HS	-	c.818G>A p.R273H	Colon	44y	F
**HUVEC primary cells**	Umbilical vein (end)	HS	-	-	Umbilical chord	-	-
**IMR32 RRID: CVCL_0346**	Neuro-blastoma (neu)	HS	-	WT	Abdomen (met)	13m	M
**LAN1 RRID: CVCL_1827**	Neuro-blastoma (neu)	HS	-	c.564C>A p.C182*	Bone marrow (met)	2y	M
**MCF7 RRID: CVCL_0031**	Adeno-carcinoma (epi)	HS	-	WT	Breast	69y	F
**MDA-MB-231 RRID: CVCL_0062**	Adeno-carcinoma (epi)	HS	-	c.839G>A p.R280K	Breast	51y	F
**NHEK primary cells**	Keratinocytes (epi)	HS	-	-	Foreskin	-	M
**Raji RRID: CVCL_0511**	Burkitt lymphoma (lym)	HS	EBV	c.638G>A; p.R213Q c.700T>C p.Y234H	Bone	11y	M
**SAOSII RRID: CVCL_0548**	Osteosarcoma (mes)	HS	-	c.?(large del) p.?	Bone	11y	F
**SKNSH RRID: CVCL_0531**	Neuro-blastoma (neu)	HS	-	WT	Bone marrow (met)	4y	F
**UD-SCC-2 RRID: CVCL_E325**	HNSCC (epi)	HS	HPV-16	WT	Hypopharynx	58y	M
**UM-SCC-3 RRID: CVCL_7740**	HNSCC (epi)	HS	-	c.743G>A p.R248Q	Nasal cavity and paranasal sinuses (LN_met)	73y	F
**UM-SCC-4 RRID: CVCL_7751**	HNSCC (epi)	HS	-	c.637C>T p.R213*	Oral tongue	48y	F
**UM-SCC-14A RRID: CVCL_7719**	HNSCC (epi)	HS	-	c.840A>T p.R280S	Floor of mouth	64y	F
**UM-SCC-22B RRID: CVCL_7732**	HNSCC (epi)	HS	-	c.659A>G p.Y220C	Hypopharynx (LN_met)	59y	F
**UM-SCC-27**	HNSCC	HS	-	c.817C>T	Oral tongue	62y	M

**RRID: CVCL_7737**	(epi)			pR273C	(LN_met)		
**UT-SCC-24A RRID: CVCL_7826**	HNSCC (epi)	HS	-	c.560_561ins49, c.560_561ins569, p.113Pro>Leu	Oral cavity; tongue	41y	M
**UT-SCC-26A RRID: CVCL_7829**	HNSCC (epi)	HS	-	c.708C>A p.Y236*	Hypopharynx (LN_met)	60y	M

RRID=research resource identifiers; end=endothelial; epi=epithelial; lym=lymphocytic; mes=mesenchymal; neu=neural; LN=lymph node; met=metastasis; “-“=no information; HS=homo sapiens; CA=chlorocebus aethiops; M=male; F=female; y=years; m=months; Information derived from Expasy, the Swiss Bioinformatics Resource Portal (https://www.expasy.org)

A

RanBP17
(orb226830)
short exposure
app. 124-125 kDa (RefSeq)

RanBP17
(orb226830)
long exposure
app. 124-125 kDa (RefSeq)

GAPDH
(sc-47724)
37 kDa

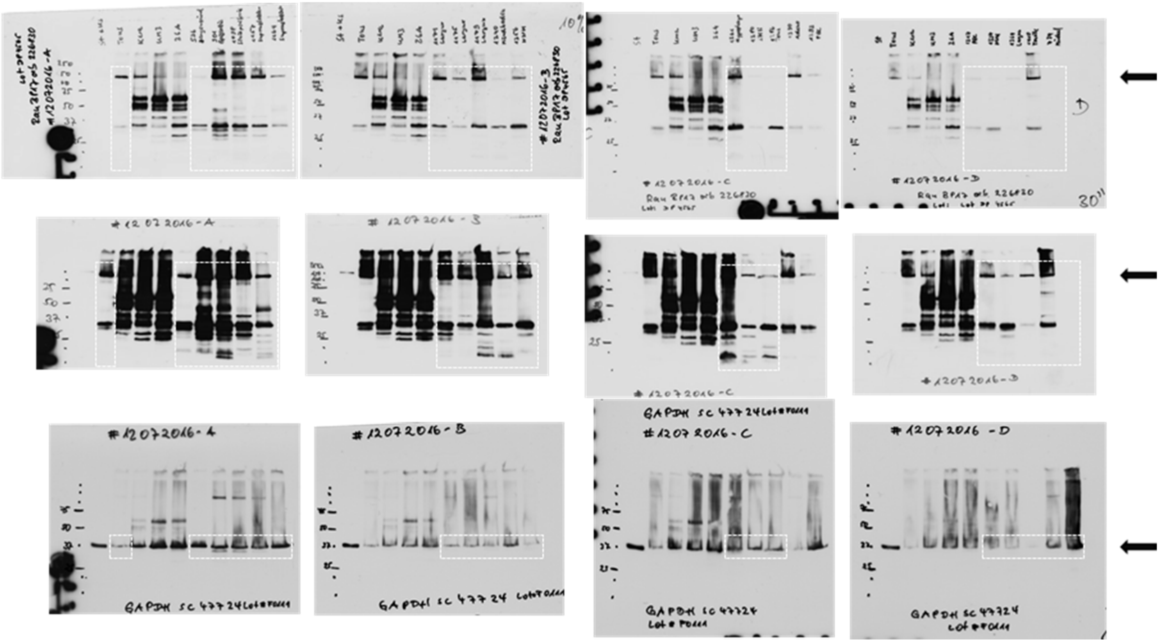

B

RanBP17
(orb226830)
short exposure
app. 124-125 kDa (RefSeq)

RanBP17
(orb226830)
long exposure
app. 124-125 kDa (RefSeq)

GAPDH
(sc-47724)
37 kDa

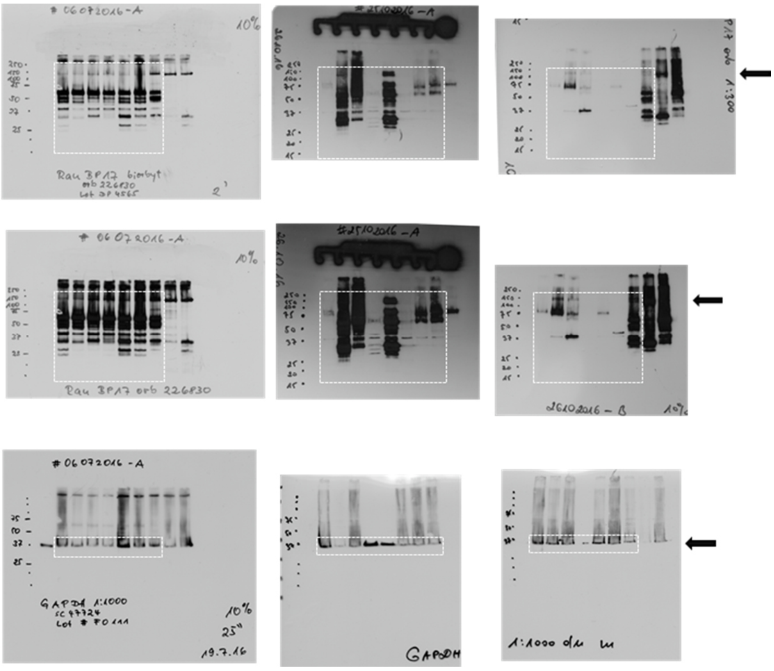

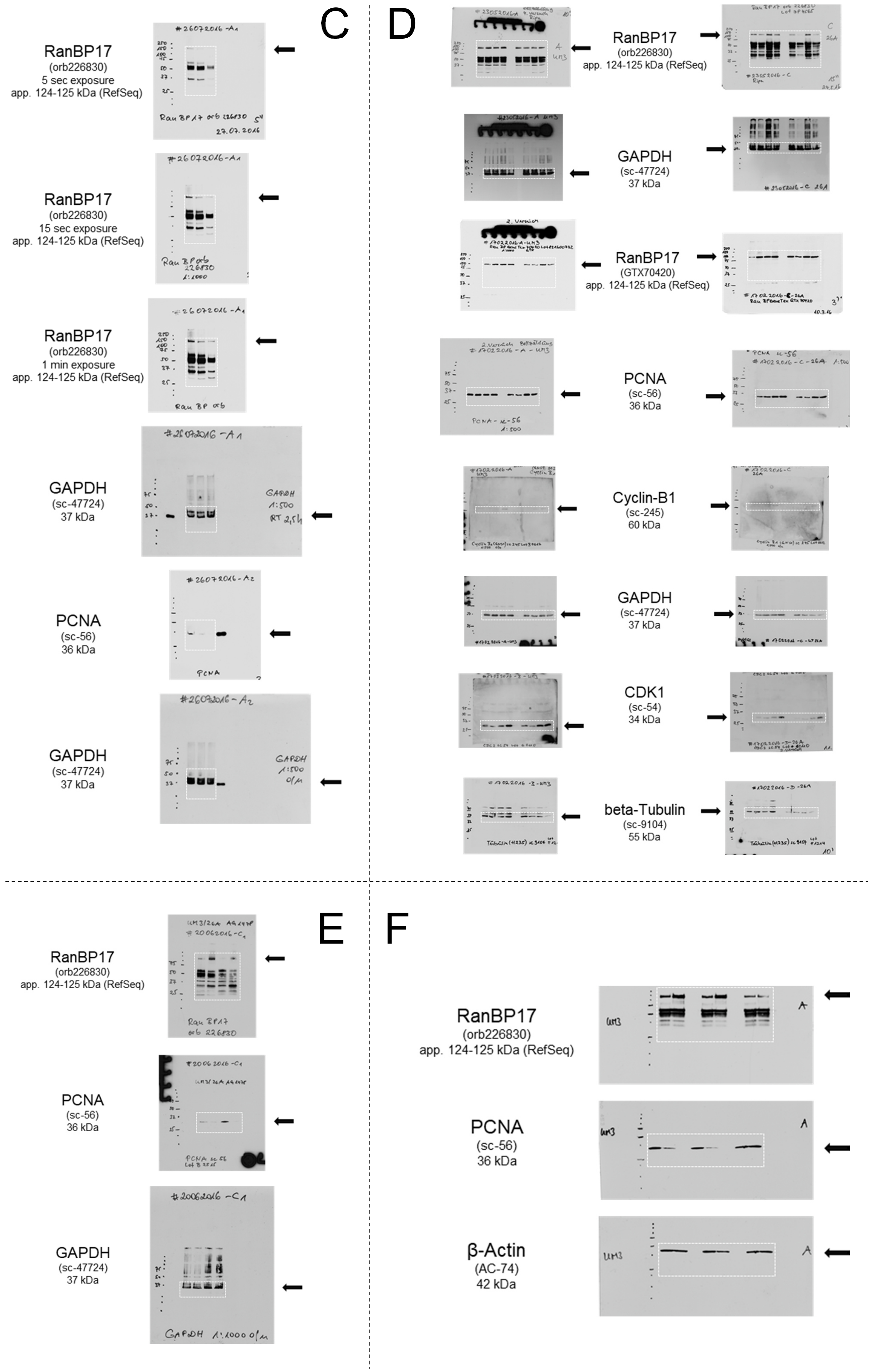

C
RanBP17
(orb226830)
5 sec exposure
app. 124-125 kDa (RefSeq)
RanBP17
(orb226830)
15 sec exposure
app. 124-125 kDa (RefSeq)
RanBP17
(orb226830)
1 min exposure
app. 124-125 kDa (RefSeq)
GAPDH
(sc-47724)
37 kDa
PCNA
(sc-56)
36 kDa
GAPDH
(sc-47724)
37 kDa
D
RanBP17
(orb226830)
app. 124-125 kDa (RefSeq)
GAPDH
(sc-47724)
37 kDa
RanBP17
(GTX70420)
app. 124-125 kDa (RefSeq)
PCNA
(sc-56)
36 kDa
Cyclin-B1
(sc-245)
60 kDa
GAPDH
(sc-47724)
37 kDa
CDK1
(sc-54)
34 kDa
beta-Tubulin
(sc-9104)
55 kDa
E
RanBP17
(orb226830)
app. 124-125 kDa (RefSeq)
PCNA
(sc-56)
36 kDa
GAPDH
(sc-47724)
37 kDa
F
RanBP17
(orb226830)
app. 124-125 kDa (RefSeq)
PCNA
(sc-56)
36 kDa
β-Actin
(AC-74)
42 kDa

Supplementary Figure S1. Uncropped Western blot images are shown for Supplementary Figure S2A (A), Supplementary Figure S2B (B), Figure 2A (C), Figure 4A (D), Figure 5A (E) and Figure 5B (F). Arrows point to the predicted size of the respective protein. Dashed rectangles refer to the region of the blot used in the final figure.

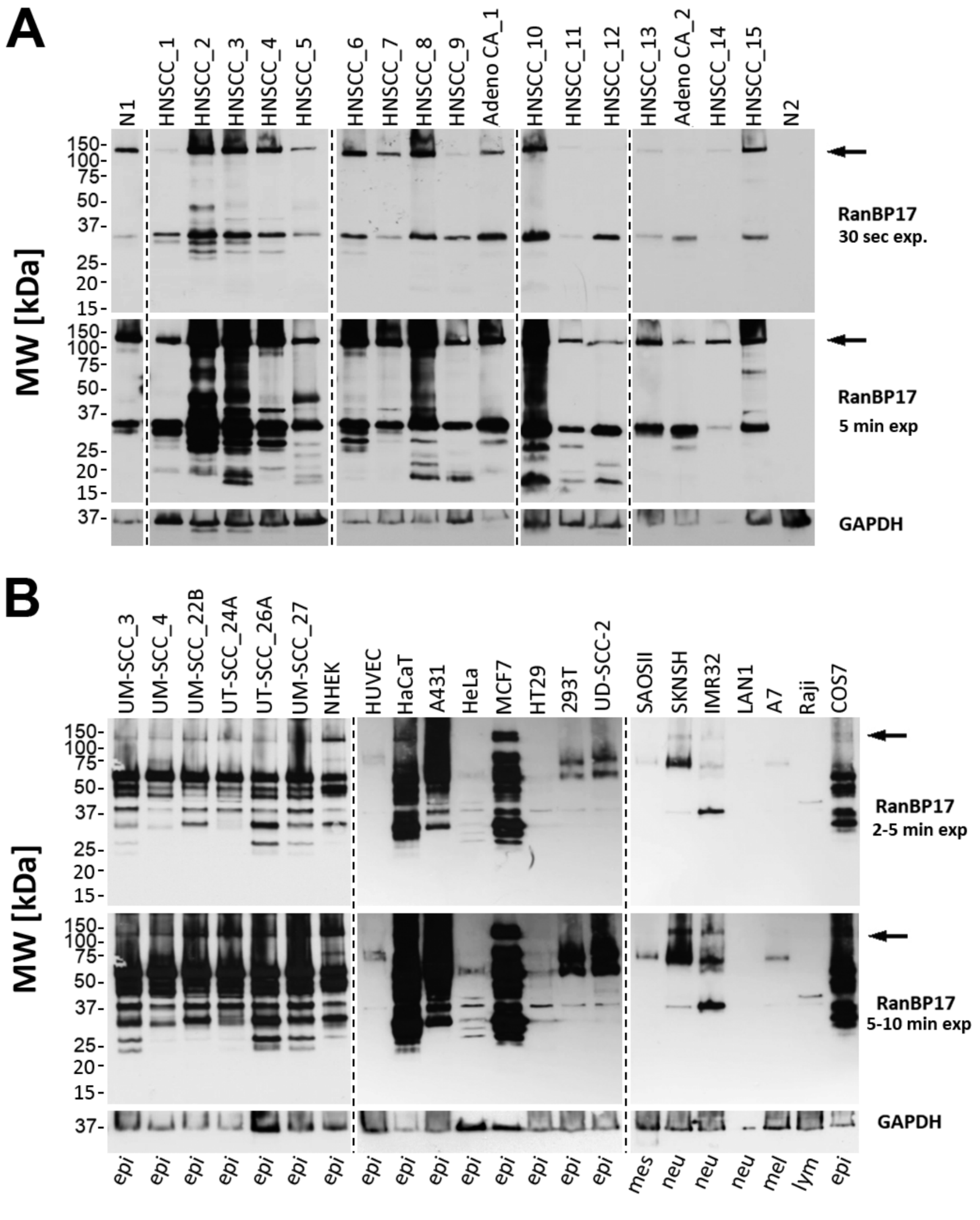

Supplementary Figure S2.

Western blot analysis, demonstrating protein expression of RanBP17 in tissues, cells and cell lines. **A**, A band at ~124 kDa (arrows) corresponding to the major RanBP17 isoform (UniProtKB/Swiss-Prot: Q9H2T7) was detected in all tested head and neck tumor tissues (HNSCC_1-15, Adeno CA_1-2) including tonsil (N1) but not muscle (N2) tissues (Supplementary Table S1). **B**, Similarly, a ~124 kDa band was also observed in all head and neck tumor derived cell lines and other cell lines derived from different tumors (Supplementary Table S2). Smaller sized immunoreactive bands are seen in most of the tested samples, especially cell lines. GAPDH was used as an input control. Short and long exposures are shown to visualize strong and weak bands. Indicated at the bottom in **B** is the histologic tumor/tissue type from which cells and cell lines were derived from. epi=epithelial, end=endothelial, mes=mesenchymal, neu=neural, mel=melanocytic, lym=lymphocytic

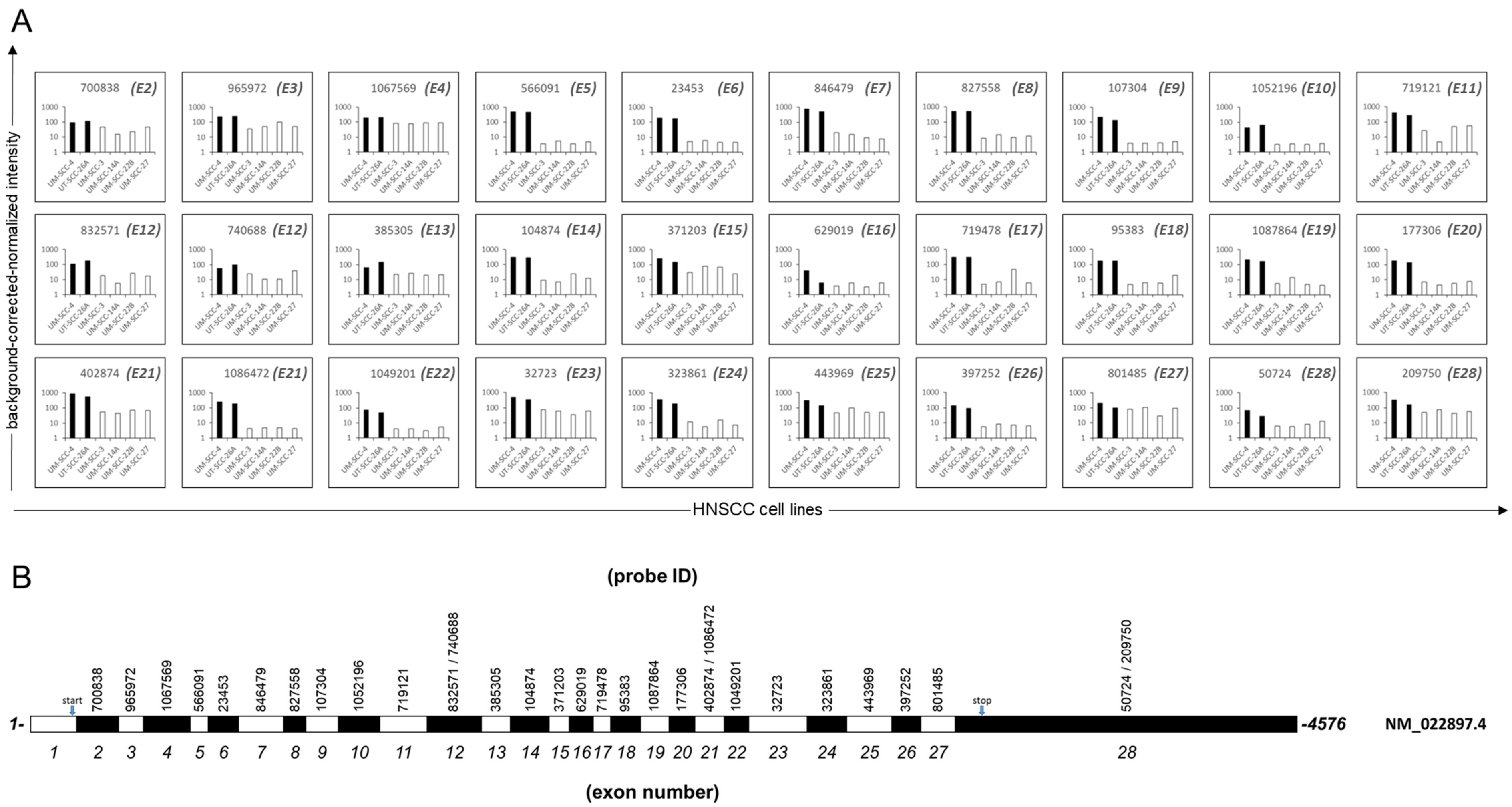

Supplementary Figure S3.

Expression of *RanBP17* exon-specific micro array probes in different HNSCC cell lines. To allow for a more precise statement about the differing *RanBP17* expression levels between the tested HNSCC cell lines during micro array analysis, we also were looking at expression of the individual exons. In order to obtain this information from the microarray files, we first searched for the sample IDs given for *RANBP17* on the Human Gene 1.0 ST Affymetrix chip used. Then, the .CEL files obtained from the microarray sequencing were imported in the program R (v. 4.0.4) using the tool oligo (v 1.54.1) and the function "read.celfiles". Afterwards, a background correction was performed using "backgroundCorrect" followed by normalization using "normalize". Finally, the obtained expression data for the previously searched *RANBP17* Probe IDs were written into a table using write.csv2 and the results were analyzed afterwards. **A**, Background corrected normalized intensity of the respective Probe signal for each tested cell line. **B**, Graphical depiction of the exon position for the single *RanBP17* specific probes according to the *RanBP17* reference sequence NM_022897.4.

Primer Pair	Primer Name	Nucleotide Sequence	Corresponding Exons	Amplicon Size (bp)	Corresponding Nucleotides in RefSeq	
					NM_022897.4	NM_022897.5
P1	rbp1-f	CTGCACTTCCAGAGTTTGGC	1	101	23-123	Not applicable
	rbp1-r	TCCAAGAGTGCTTTCTCAGCC	1			
P2	rbp2-f	TGTCAGCCGAGTCAGTCCTT	2	106	226-331	90-195
	rbp2-r	TTGGATGACAAAGGGAGCCAG	3			
P3	rbp3-f	ATTACGTGGCATCACAGCCC	2	100	288-387	152-251
	rbp3-r	TTCTGAACCTCAAACCACCCC	3			
P4	rbp4-f	AGCTGGCTCCCTTTGTCATC	2	150	309-458	173-322
	rbp4-r	TGCAGTGTTCCACAGTACCC	4			
P5	rbp5-f	GCAGGTCTTGAAACTGGTCCT	6	102	664-765	528-629
	rbp5-r	GTTGTTGGAATCTGCACCGT	7			
P6	rbp6-f	GCACCAGAAATCACGAAGGC	10	128	1229-1356	1093-1220
	rbp6-r	GTGCACAACTGCTCCAACTG	11			
P7	rbp7-f	GACGTCTTGCATGGCTGGTA	12	150	1485-1634	1349-1498
	rbp7-r	ATCGAGGCAATCCGGTATCC	13			
P8	rbp8-f	TGCTGCCTCTTACAGTTGCT	18	107	2085-2191	1949-2055
	rbp8-r	TCGAAGATCTCTTGCCAGCC	18, (19)			
P9	rbp9-f	AGGGATTGCCTTTGCACTGA	19	114	2191-2304	2055-2168
	rbp9-r	GGCTCTCCATACCACCGTTC	20			
P10	rbp10-f	GCAGCAAAACCCAGATGTCC	25	123	2932-3054	2796-2918
	rbp10-r	TCATTGAGCAGGATGAGCCC	25			
P11	rbp11-f	ATGTGCGGAGCAGCCTTTAT	27	115	3300-3414	3164-3278
	rbp11-r	AGGGCTTTTGCTGTTGCTTG	28			
**Primer Pair**	**Primer Name**	**Nucleotide Sequence**	**Corresponding Exons**	**Amplicon Size (nt)**	**Corresponding Nucleotides in RefSeq**	
					**NM_002046.7**	**Not applicable**
GAPDH	gapdh-f	CCTCCTGTTCGACAGTCAGC	1	314	11-324	Not applicable
	gapdh-r	GAGGGATCTCGCTCCTGGAA	4, 5			
RPLPO	rplp0-f	CGTCCTCGTGGAAGTGACAT	1, 2	246	16-261	Not applicable
	rplp0-r	GCATCATGGTGTTCTTGCCC	3			

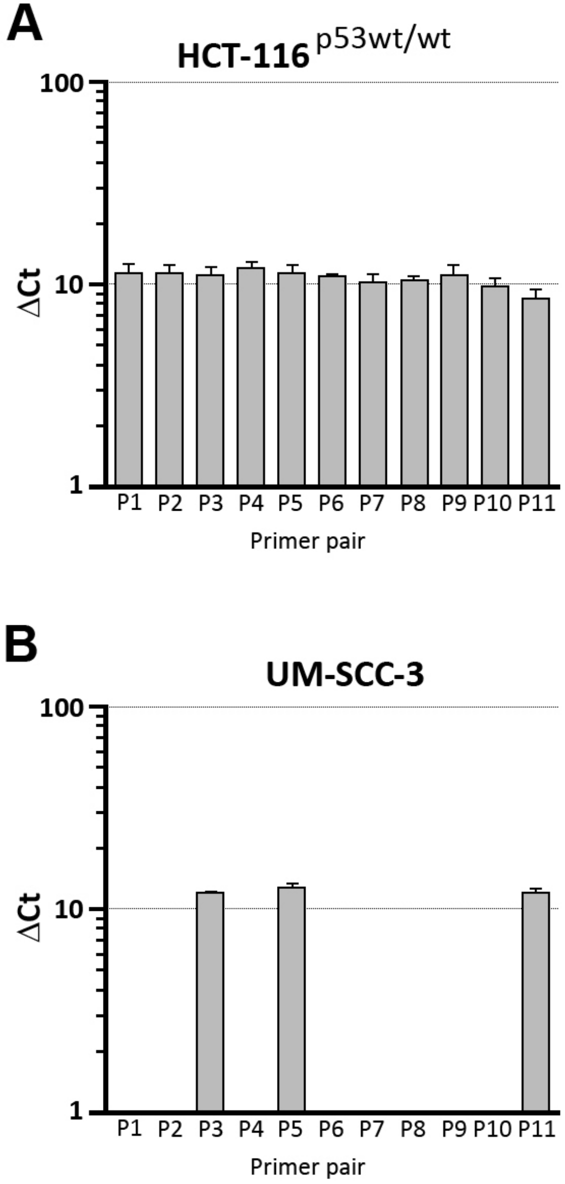

Supplementary Figure S4.

Shown on the left are the primer pairs used for the quantitative PCR analysis in Fig. 3 with their corresponding sequence information. **A** and **B** show comparable efficiencies of all primer pairs in **A**) untreated HCT-116$^{p53wt/wt}$ cells and **B**) untreated UM-SCC-3 cells. In UM-SCC-3 cells, only primer pairs P3, P5, and P11 yielded amplicons.

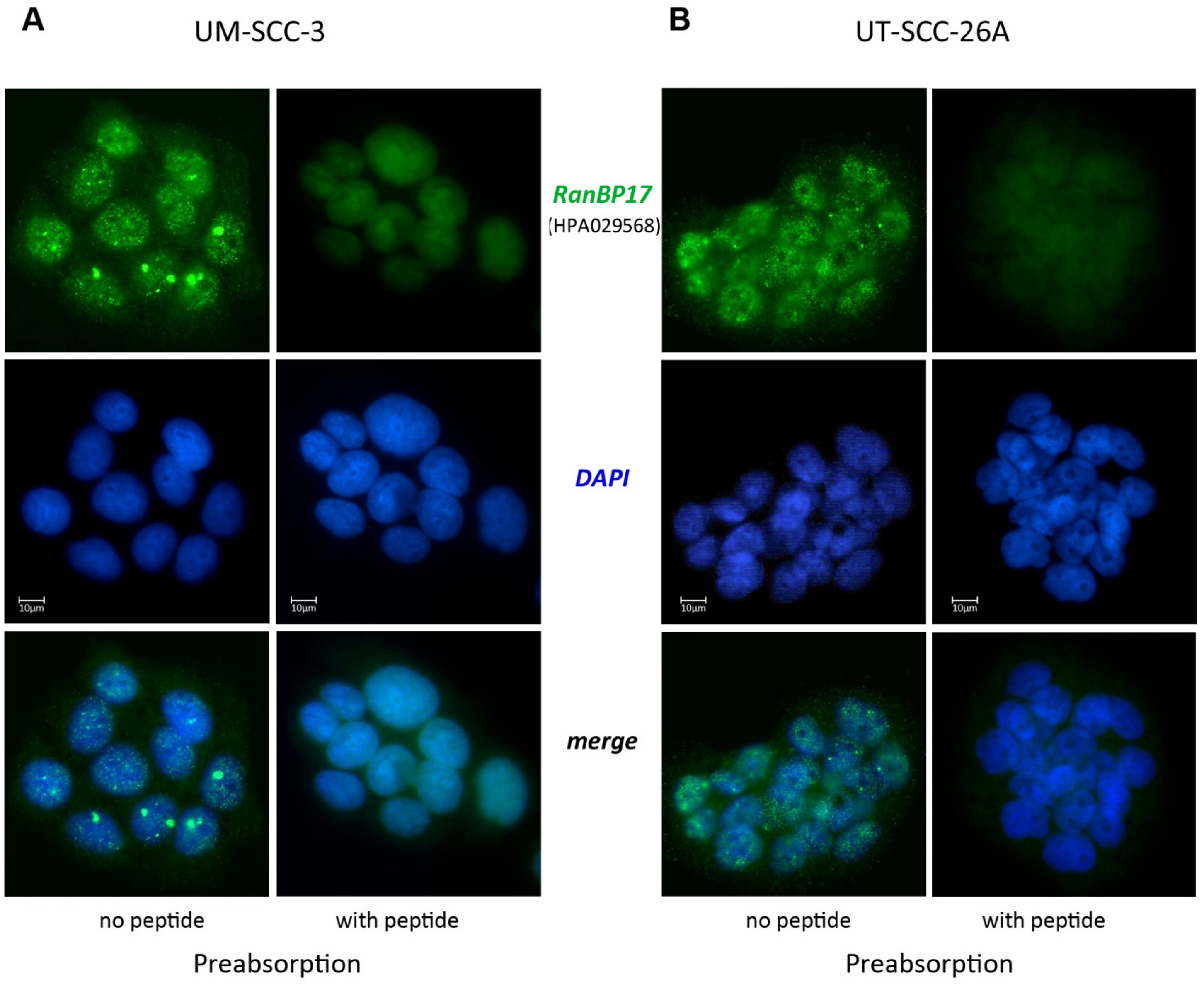

Supplementary Figure S5.

Specificity of the RanBP17 antibody. The RanBP17 signal (left panel in **A, B**) disappears (right panel in **A, B**) after preincubating the RanBP17 antibody (HPA029568) with its corresponding antigen peptide sequence (APrEST73986) thereby confirming specificity of the antibody. *TP53* mutation in UM-SCC-3: p.R248Q (nuclear p53); *TP53* mutation in UT-SCC-26A: p.Y236* (cytoplasmic p53).

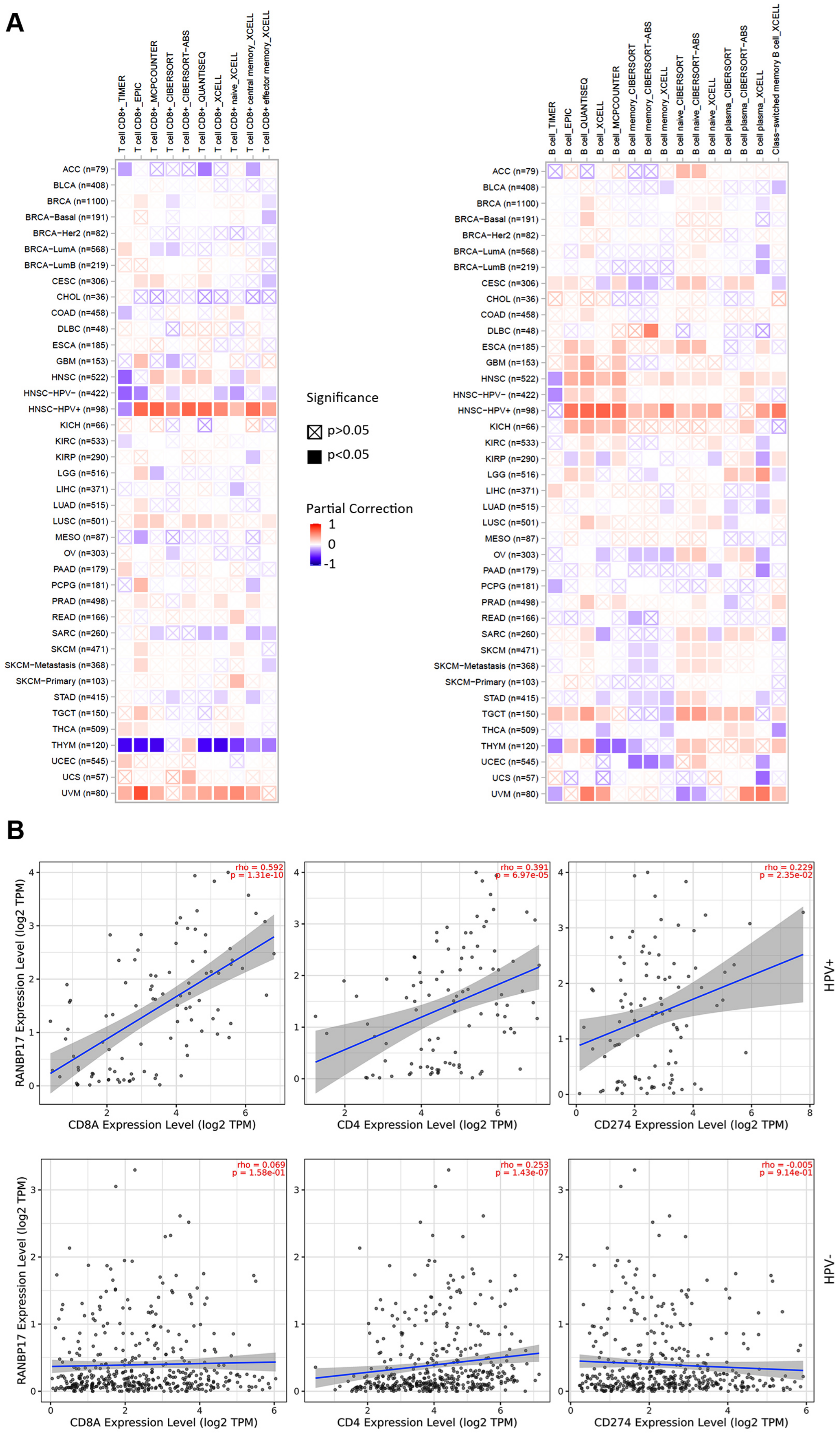

Supplementary Figure S6.

Timer2.0 (http://timer.cistrome.org/) was used for the analysis of **A**, the immune association for *RANBP17* and CD8+ T-cells (left side) and B-cells (right side) showing the Spearman correlation coefficients. Color filled boxes indicate significantly higher values ($p<0.05$) and **B**, for the correlation between the *RANBP17* expression to CD8A/CD4/CD274 for HPV+ (upper part) and HPV- (lower part) samples of the TCGA-HNSC cohort (accessible at Gene_Corr of the Exploration tab), with the blue depicting the linear regression and the grey shaded area depicting the confidence interval. TPM: transcripts per kilobase million.

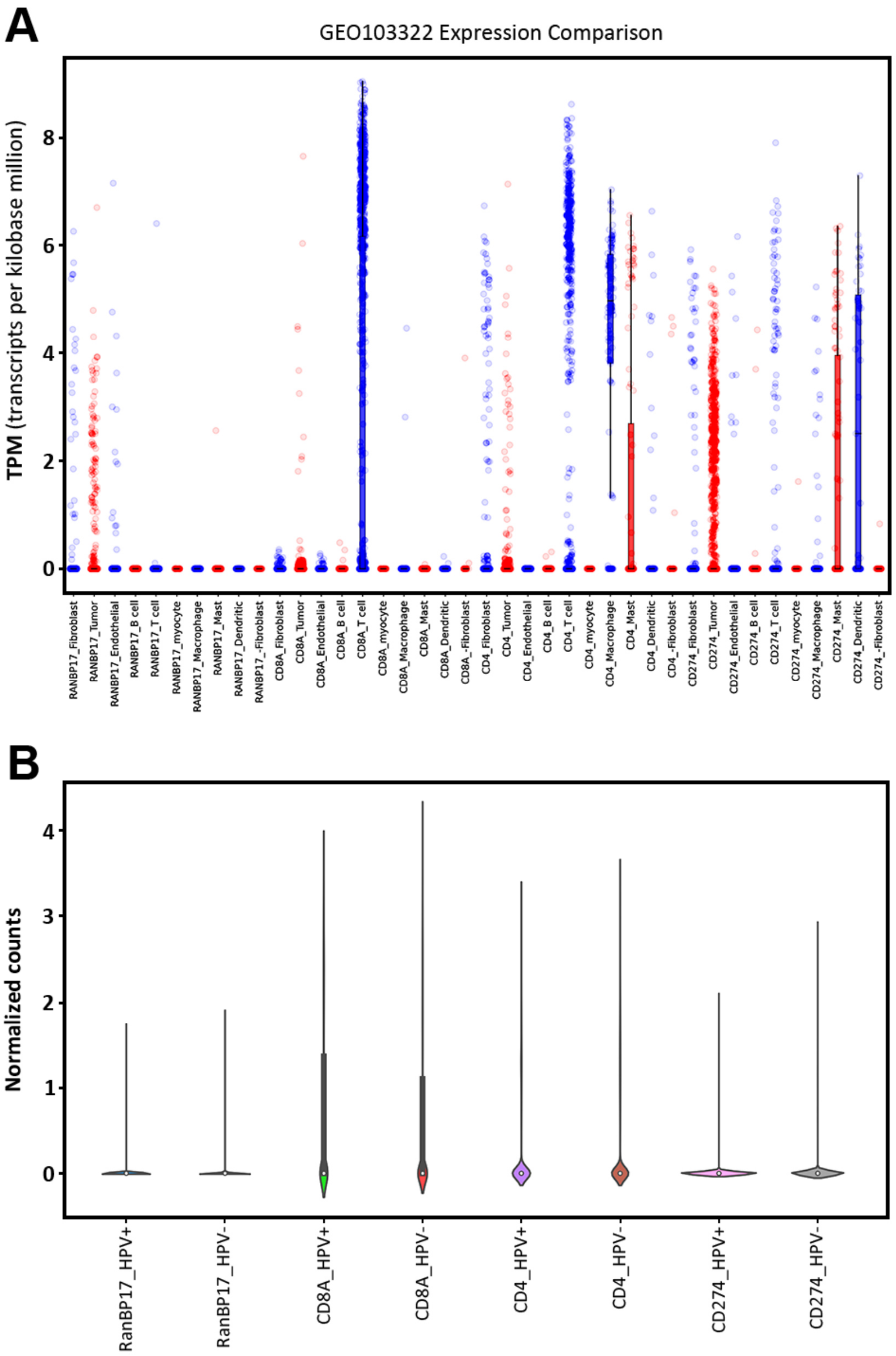

Supplementary Figure S7.

A, Boxplots for a scRNA-seq (single cell RNA-sequencing) HNSCC dataset (GEO103322) showing the expression of *RANBP17*, *CD8a*, *CD4*, and *CD274* for the different cell types. Each dot represents the expression of one sequenced cell. **B**, Violin plots for the scRNA-seq immune cell HNSCC dataset (GEO139324), for which the expression details for each cell were extracted from the provided matrix files using Seurat for R. Plots were generated using the modules matplotlib and seaborn for python.

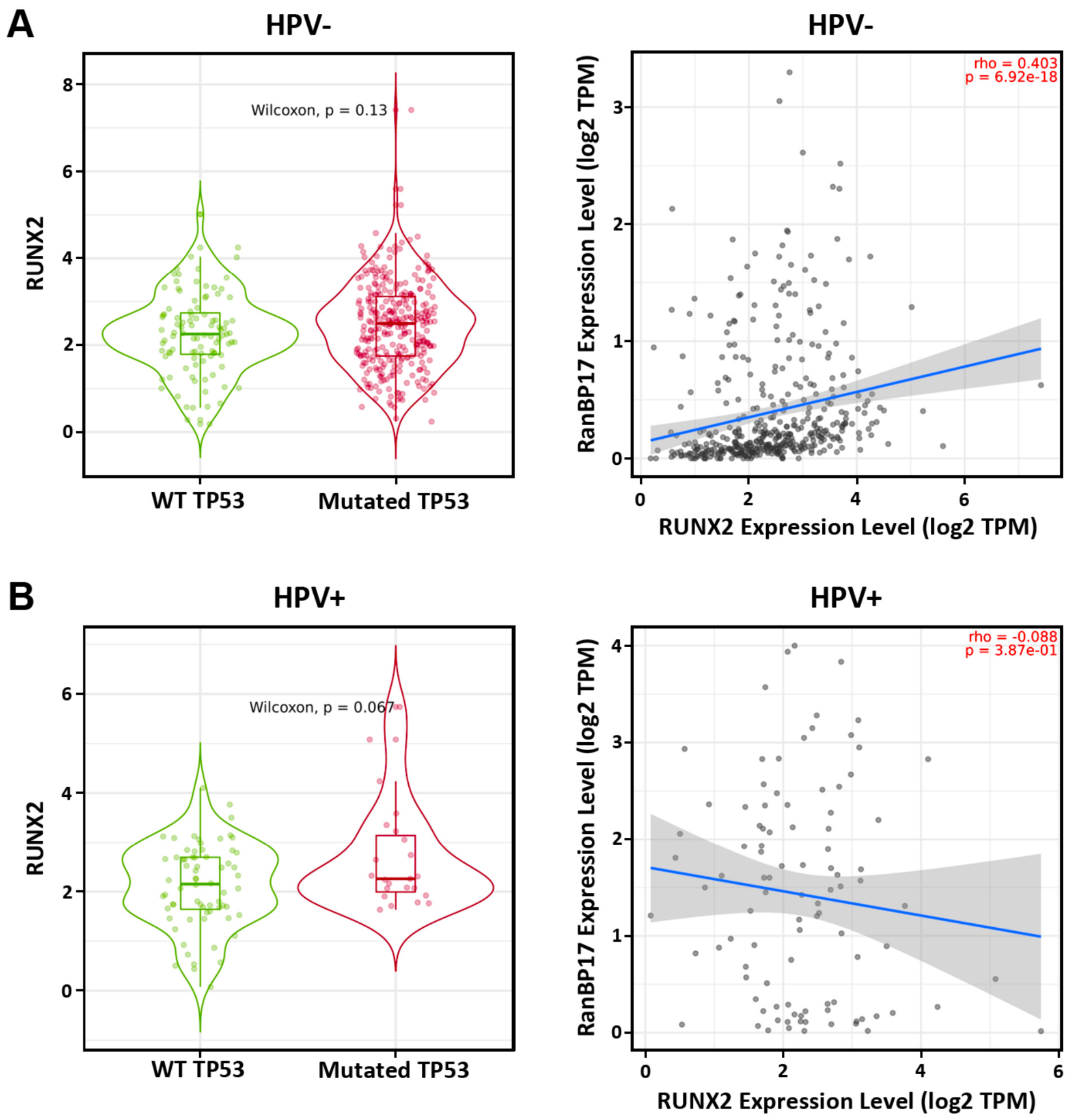

Supplementary Figure S8.

Boxplot analysis of the *TP53* mutational status with regard to *RUNX2* expression as well as correlation analysis of *RUNX2* with *RanBP17* for the **A**, HPV- and **B**, HPV+ TCGA-HNSCC cohorts, showing no significant expression differences with regard to *TP53* mutational status but a significant correlation in the HPV- cohort only. Analyses were performed using the TIMER2.0 Gene_Mutation module (http://timer.cistrome.org/). Left graphs: Wilcoxon test. Right graphs: linear regression with Spearman's rho.
